# circ_PPAPDC1A promotes Osimertinib resistance by sponging the miR-30a-3p/ IGF1R pathway in non-small cell lung cancer (NSCLC)

**DOI:** 10.1186/s12943-024-01998-w

**Published:** 2024-05-07

**Authors:** Yi-fang Tang, Zheng-hua Liu, Lei-yi Zhang, Sheng-hao Shi, Shun Xu, Jin-An Ma, Chun-Hong Hu, Fang-wen Zou

**Affiliations:** 1 Department of Anesthesiology, The Second Xiangya Hospital of Central South University, Changsha, 410000 Hunan P.R. China; 2 Department of Thoracic Surgery, The First Affiliated Hospital of China Medical University, Shenyang, 11000 Liaoning P.R. China; 3 Department of General Surgery, The Second Xiangya Hospital of Central South University, Changsha, 410000 Hunan P.R. China; 4 Department of Oncology, The Second Xiangya Hospital of Central South University, Changsha, 410000 Hunan P.R. China

**Keywords:** NSCLC, Osimertinib resistance, circ_PPAPDC1A, miR-30a-3p, IGF1R

## Abstract

**Background:**

Recent evidence has demonstrated that abnormal expression and regulation of circular RNA (circRNAs) are involved in the occurrence and development of a variety of tumors. The aim of this study was to investigate the effects of circ_PPAPDC1A in Osimertinib resistance in NSCLC.

**Methods:**

Human circRNAs microarray analysis was conducted to identify differentially expressed (DE) circRNAs in Osimertinib-acquired resistance tissues of NSCLC. The effect of circ_PPAPDC1A on cell proliferation, invasion, migration, and apoptosis was assessed in both in vitro and in vivo. Dual-luciferase reporter assay, RT-qPCR, Western-blot, and rescue assay were employed to confirm the interaction between circ_PPAPDC1A/miR-30a-3p/IGF1R axis.

**Results:**

The results revealed that circ_PPAPDC1A was significantly upregulated in Osimertinib acquired resistance tissues of NSCLC. circ_PPAPDC1A reduced the sensitivity of PC9 and HCC827 cells to Osimertinib and promoted cell proliferation, invasion, migration, while inhibiting apoptosis in Osimertinib-resistant PC9/OR and HCC829/OR cells, both in vitro and in vivo. Silencing circ_PPAPDC1A partially reversed Osimertinib resistance. Additionally, circ_PPAPDC1A acted as a competing endogenous RNA (ceRNA) by targeting miR-30a-3p, and Insulin-like Growth Factor 1 Receptor (IGF1R) was identified as a functional gene for miR-30a-3p in NSCLC. Furthermore, the results confirmed that circ_PPAPDC1A/miR-30a-3p/IGF1R axis plays a role in activating the PI3K/AKT/mTOR signaling pathway in NSCLC with Osimertinib resistance.

**Conclusions:**

Therefore, for the first time we identified that circ_PPAPDC1A was significantly upregulated and exerts an oncogenic role in NSCLC with Osimertinib resistance by sponging miR-30a-3p to active IGF1R/PI3K/AKT/mTOR pathway. circ_PPAPDC1A may serve as a novel diagnostic biomarker and therapeutic target for NSCLC patients with Osimertinib resistance.

**Graphical Abstract:**

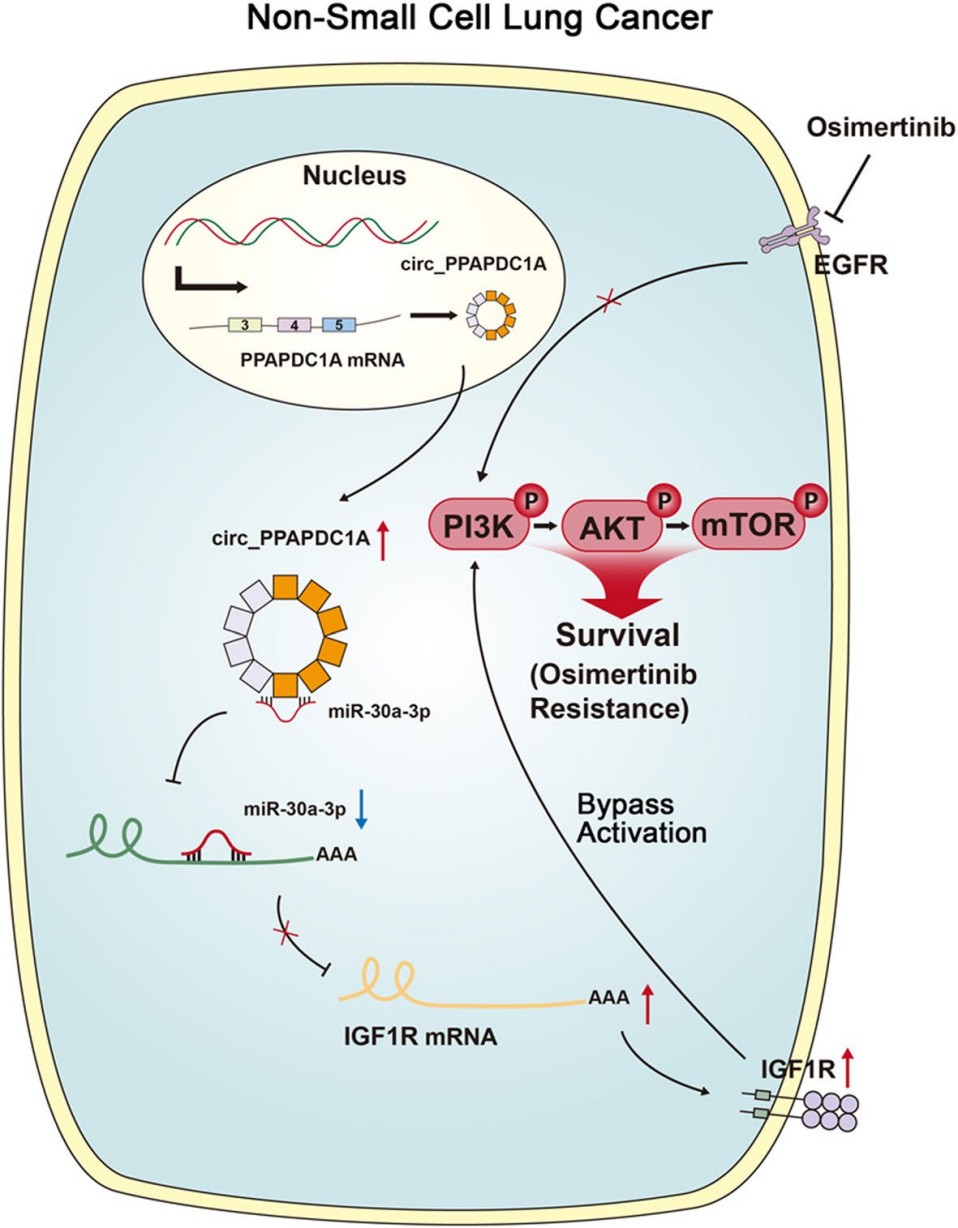

**Supplementary Information:**

The online version contains supplementary material available at 10.1186/s12943-024-01998-w.

## Introduction

Non-small cell lung cancer (NSCLC) is the most common type of lung cancer, but over 70% of patients are already in locally advanced or metastatic stages at the time of diagnosis, depriving them of the opportunity for curative surgical treatment and making it a leading cause of cancer-related deaths worldwide [1, 2]. With in-depth research on lung cancer genotyping and the rapid development of molecular targeted therapies, targeted therapy has become the standard first-line treatment for advanced NSCLC patients with driver gene mutations (such as EGFR and ALK) [3, 4]. Targeted therapy has ushered in the era of precision medicine in the diagnosis and treatment of NSCLC [4, 5].

Osimertinib, a third-generation EGFR-TKIs, effectively targets EGFR mutations and the T790M resistance mutation [6]. It’s the preferred first-line therapy for EGFR-positive NSCLC patients, as proven by the FLAURA trial [7, 8]. However, patients who initially respond to Osimertinib have a median progression-free survival of approximately 18.9 months, and after second-line treatment, they typically develop resistance after about 10 months [9]. Despite the initial success of targeted therapies, Osimertinib- resistance remains a significant challenge. Various known mechanisms include EGFR mutations (G719X, G796X, C797S) and wild-type EGFR amplification [10]. EGFR-independent factors like histological changes, alternative pathways (c-Met, HER2 amplification, Activation of IGF1R), NRAS/KRAS mutations, and oncogenic fusion genes (RET, ALK, BRAF) also contribute [11]. Still, around 40-50% of resistance mechanisms remain unclear [12]. Understanding Osimertinib resistance is crucial for improving treatment and patient outcomes.

Circular RNAs (circRNAs) are unique RNA molecules with closed loops, formed by back-splicing of pre-mRNAs [13]. Initially seen as splicing errors, circRNAs are now recognized as vital gene expression regulators. Functional circRNAs were initially found to efficiently sponge microRNAs, thereby regulating downstream target genes post-transcriptionally. They also serve as reservoirs or carriers for miRNAs [14]. This phenomenon is substantiated by the discovery of cerebellar degeneration-related protein (CDR1as), also known as CiRS-7 [15], opened the door to the understanding that circRNAs can compete as endogenous RNAs (ceRNA) with over 60 miRNA binding sites. Subsequent studies have further affirmed circRNAs’ role as ceRNA or miRNA sponges. The regulation of circRNAs also transitions the traditional miRNA→mRNA→protein model to a new regulatory model of circRNA→miRNA→mRNA→protein. In this new regulatory model, circRNAs communicate with each other using miRNA response elements (MREs) as a language, competing to regulate the expression of other miRNAs that share the same MREs [16]. Individual miRNAs can, in turn, regulate the expression levels of multiple mRNAs, and a single gene can be simultaneously regulated by multiple miRNAs, thus forming a vast interconnected regulatory network that modulates gene expression at the post-transcriptional level [17]. This network influences the occurrence, development, and progression of various diseases [18].

Current research suggests that circular RNA (circRNAs) exhibits abnormal expression in non-small cell lung cancer (NSCLC) and is closely associated with processes such as tumor proliferation, invasion, and metastasis. Some circRNAs are significantly upregulated or downregulated, playing an important role in regulating gene expression during the development of lung cancer [19]. Additionally, circRNAs also participates in modulating NSCLC’s resistance to chemotherapy and radiotherapy [20]. In the context of EGFR-TKIs treatment, circRNAs has also been implicated in the development of resistance. Notably, in EGFR-TKIs resistant patients, certain circRNAs show significant upregulation [21]. These circRNAs may influence the effectiveness of drugs by modulating the EGFR pathway and other critical signaling pathways, contributing to the failure of EGFR-TKIs treatment [22].

Overall, circRNAs plays a crucial regulatory role in the occurrence, development, chemotherapy resistance, and EGFR-TKIs resistance of lung cancer. In-depth investigation into the functions and regulatory mechanisms of circRNAs in NSCLC could reveal the molecular basis of cancer development and drug resistance, providing novel strategies and targets for the diagnosis and treatment of NSCLC, offering hope for patient prognosis and survival. However, the correlation between circRNAs and Osimertinib resistance in NSCLC, along with its functional roles and mechanisms, remains poorly studied. This research aims to explore the impact of circRNAs on Osimertinib resistance in NSCLC and its underlying mechanisms, which holds important clinical significance in understanding the molecular mechanisms of NSCLC Osimertinib resistance and devising strategies to delay or overcome resistance.

## Materials and methods

### Tissues collected

Five cases of non-small cell lung cancer (NSCLC) tissues, consisting of patients who were sensitive to Osimertinib (Osimertinib Sensitive, OS), as well as the corresponding cancer tissues from these patients after developing Osimertinib-acquired resistance (Osimertinib Resistance, OR), selected as the subjects for this study, were collected from The First Affiliated Hospital of China Medical University, and approved by the institutional review board of Medical Ethics Committee of Xiangya Second Hospital, Central South University (approval No.2018016). All five patients received first-line treatment with Gefitinib (with EGFR-sensitive mutations before treatment), and second-line treatment with Osimertinib. Clinical efficacy assessment (RECIST 1.1) [23] and gene testing results [24] were comprehensively considered to determine Osimertinib sensitivity and acquired resistance. The selection criteria for Osimertinib-sensitive patients were as follows: patients with EGFR exon 20 T790M secondary mutations, who showed partial response (PR) or complete response (CR) after one month of Osimertinib treatment. On the other hand, the criteria for Osimertinib-resistant patients were the presence of EGFR C797S point mutation, T790M deletion, c-MET amplification, and apparent disease progression (PD) upon clinical assessment during Osimertinib treatment. The study was approved by the institutional review board of Medical Ethics Committee of Xiangya Second Hospital, Central South University (approval No.2022324). And the methods and procedures for patient specimen collection comply with the relevant regulations and requirements of the Medical Ethics Committee in accordance with the Declaration of Helsinki [25], and informed consent was obtained from all patients. The basic clinical and pathological data of five enrolled patients are detailed in Supplemental Table 1.

### Human circRNAs microarray analysis

Collect NSCLC tissues before and after Osimertinib resistance, extract RNA, and check its quality. Transcribe RNA into cDNA using reverse transcriptase with a reverse primer to distinguish linear and circular RNA (Epicentre; Illumina, Inc.). Enrich circular RNA with RNase R enzyme, preserving it while degrading linear RNA (Invitrogen; Thermo Fisher Scientific, Inc.). Label and hybridize circular RNA with fluorescent probes on a circRNA chip (Agilent Technologies, Inc.). After washing, laser scan the chip’s signals. Analyze data bioinformatically to identify differentially expressed circRNAs before and after Osimertinib resistance. Heatmap Generation: Generate a heatmap to visualize the expression patterns of differentially expressed circRNAs across samples. Use hierarchical clustering or other clustering algorithms to group samples and circRNAs based on similarity in expression profiles. Color code the heatmap to represent expression levels, with upregulated circRNAs in red color and downregulated circRNAs in green color. Scatter Plots: Generate scatter plots to compare the expression levels of individual circRNAs between experimental groups. Plot the expression values of circRNAs in one group against those in another group, with each data point representing a circRNA. Highlight differentially expressed circRNAs with distinct colors or shapes for easy visualization. Volcano Plots: Create volcano plots to simultaneously visualize fold changes and statistical significance of differentially expressed circRNAs. Plot the log2(fold change) on the x-axis and the -log10(*p*-value) on the y-axis. Highlight significantly upregulated circRNAs in red color, significantly downregulated circRNAs in green color, and non-significant circRNAs in a neutral color. GO Classes Bar Plots: Perform gene ontology (GO) analysis to identify enriched biological processes, cellular components, and molecular functions associated with differentially expressed circRNAs. Generate bar plots to visualize the enriched GO classes and their significance levels. Plot the -log10(*p*-value) or enrichment score on the y-axis and the GO terms on the x-axis. Pathway Enrichment Plots: Conduct pathway enrichment analysis to identify signaling pathways and biological pathways enriched with differentially expressed circRNAs. Create pathway enrichment plots to visualize the enriched pathways and their significance levels. Plot the -log10(*p*-value) or enrichment score on the y-axis and the pathway names on the x-axis. Please refer to the Supplemental Human Circular RNA Microarray Report for detailed.

### Bioinformatics data curation, and normalization [26, 27]

Data preprocessing: Prior to bioinformatics analysis, raw data undergo preprocessing, including data cleaning, format conversion, and removal of invalid information. This can be achieved using specialized data processing software or programming languages. Quality control: Raw data undergo quality control, including examination of quality metrics such as signal intensity, background noise, probe quality, etc., and exclusion of low-quality samples or probes to ensure the reliability of subsequent analyses. Normalization: Data normalization is performed on high-quality data to eliminate technical variations between different samples. Common normalization methods include quantile normalization, Z-score normalization, etc., to ensure comparability of data across different samples. Differential expression analysis: Statistical methods are employed to compare differential expression between different sample groups and identify differentially expressed circRNAs. This can be accomplished using statistical tests such as t-tests, ANOVA, linear models, etc. Data integration and annotation: Differentially expressed circRNAs are compared with known circRNA databases, and bioinformatics annotations, including enrichment analysis, pathway analysis, etc., are conducted to further understand their functions and regulatory mechanisms in biological processes. Data visualization: Various visualization tools and techniques, including heatmaps, volcano plots, scatter plots, etc., are used to visualize analysis results and illustrate the relationships between differentially expressed circRNAs. Data storage and sharing: Analysis results are stored in databases or online platforms, and consideration is given to sharing data with other researchers to promote research reproducibility and scientific exchange.

### Cells

Human normal lung epithelial cells EAS-2B, The Osimertinib sensitive NSCLC cell lines PC9 and HCC827 were obtained from the American Type Culture Collection (ATCC) and maintained in 10% fetal bovine serum (Invitrogen; Thermo Fisher Scientific, Inc.) with Dulbecco’s modified Eagle’s medium (Invitrogen; Thermo Fisher Scientific, Inc.) at 37 °C, 95% O_2_ and 5% CO_2_. Additionally, the Osimertinib-resistant cell lines, PC9/OR (Osimertinib-resistant PC9 cells) and HCC827/OR (Osimertinib-resistant HCC827 cells), were generously provided by Dr. Liu from China Medical University. PC9/OR and HCC827/OR cell lines were established by subjecting PC9 and HCC827 cells to long-term drug stimulation and selection under specific culture conditions to obtain stable resistant cell lines. PC9 and HCC827 cell lines harbor EGFR exon 19 E746-A750 deletion mutations, while PC9/OR and HCC827/OR cells exhibit c-MET gene amplification (Shanghai Ranstone Medical Co., Ltd.).

### Reverse transcription-quantitative (RT-q) PCR

Total RNA was extracted using TRIzol® reagent (Invitrogen; Thermo Fisher Scientific, Inc.). Subsequently, cDNA was synthesized with the PrimeScript™ RT-PCR Kit (cat. no. 4368814; Invitrogen; Thermo Fisher Scientific, Inc.), following the manufacturer’s protocol. The Thermal Cycler Dice System (Takara Bio, Inc.) with the SYBR Green PCR Master Mix (Thermo Fisher Scientific, Inc.) The primer sequences for circRNAs, miRNA, and mRNA were custom-synthesized by Shanghai GeneChem Co., Ltd. GAPDH was utilized as the internal reference for circRNAs and mRNA, while U6 served as the internal reference for miRNA. The thermocycling conditions involved an initial step at 95 °C for 15 min, followed by 40 cycles at 94 °C for 15 s, 55 °C for 30 s, and 70 °C for 30 s. Following the PCR procedure, the relative expression levels were calculated using the 2^−ΔΔCq^ method. Experiment repeated thrice. Please refer to the Supplemental Materials and Methods for detailed.

### Cell Counting Kit-8 (CCK-8)

Cells in logarithmic growth were trypsinized, suspended in 5 mL 1640 medium, and adjusted to 1 × 10^4^ cells/mL. 2000 cells/well were seeded in a 96-well plate with 200 µL PBS as control. AZD9291 (0.0–1.0 µM) was added to each group with 6 replicates. After 24 h, 10 µL CCK-8 solution (cat. no. ab228554; Abcam Biotech Co., Ltd.) was added, incubated for 4 hours, followed by 150 µL DMSO (Sigma-Aldrich Co., Ltd.). Absorbance (OD490) was measured at 492 nm using a microplate reader (Bio-Tek Instruments, Inc.). Cell viability was calculated using the formula: Cell Viability = (Exp. group OD490-Blank control OD490) / (Neg. control OD490 - Blank control OD490) × 100%. Experiment repeated thrice. Please refer to the Supplemental Materials and Methods for detailed.

### Soft agar colony formation assay

Cells in logarithmic growth phase were digested with 0.25% trypsin, counted, and adjusted to 1 × 10^4^ cells/mL. Preparation of semi-solid agar (cat. no. HB8897; Qingdao Haibo Bio, Co., Ltd.), divided into bottom and top layers. Bottom layer agar: Weigh 0.25 g of agar and add 32 mL of double-distilled water. After autoclaving, cool to 50 °C, then sequentially add 8 mL of preheated RPMI-1640 medium (5x concentrated) at 50 °C, 10 mL of fetal bovine serum, 100 U/mL penicillin, 100 µg/mL streptomycin, and 0.2 mL of 8% NaHCO3. Mix well and distribute 3 mL per 60 mm culture dish. Allow to solidify at room temperature. Top layer agar: Weigh 0.15 g of agar and add 32 mL of double-distilled water. After autoclaving, cool to 40 °C, then add the same components as the bottom layer. Mix well and keep warm in a 40 °C water bath to prevent solidification. Take 0.1 mL of cell suspension and add it to 5 mL of 40 °C top layer agar solution. Mix well and quickly add 0.5 mL to the culture dish with the bottom layer agar. Allow to solidify at room temperature for 10 min, then transfer to a CO2 incubator. After 44 h, add 0.5 µM AZD9291 every 3 days and culture at 37 °C for 2 weeks. When visible colonies form, transfer to a 6-well plate, discard the culture medium, fix with 2 mL of methanol per well for 30 min, discard methanol, stain with 2 mL of 0.1% crystal violet (Invitrogen; Thermo Fisher Scientific, Inc.) for 3 min, then rinse off the crystal violet, photograph with a digital camera, and count the colonies under a light microscope (Olympus Corporation). Please refer to the Supplemental Materials and Methods for detailed.

### EdU staining assay

Cells in logarithmic growth phase were digested and resuspend cells in complete medium to a density of 2 × 10^5 cells/ml. Prepare a 6-well plate and seed 100 µL of cell suspension into each well. Incubate overnight at 37 °C in a constant temperature cell culture incubator until cells adhere to the bottom. Treat each group of cells with 0.5 µM AZD9291. Dilute EdU solution (cat. no. A10044; Thermo Fisher Scientific, Inc.) (Reagent A) with cell culture medium at a ratio of 1000:1 to prepare a sufficient amount of 50µM EdU culture medium (EdU labeling). Add 100 µl of diluted EdU working solution to each well to achieve a final concentration of 10 µM. Incubate for 2 h, then fix cells with 50 µL of 4% paraformaldehyde (cat. no. 30525-89-4; Sigma-Aldrich, Inc.) at room temperature for 30 min. Stain with Hoechst33342 (cat. no. 23491-45-4; Sigma-Aldrich, Inc.) in the dark for 10 min. Observe EdU-labeled cells under a fluorescence microscope, photograph, and count. Experiment repeated thrice. Please refer to the Supplemental Materials and Methods for detailed.

### Matrigel invasion assay

Cells in logarithmic growth phase were digested and resuspend cells in complete medium to a density of 2 × 10^5 cells/ml. Thaw Matrigel overnight at 4 °C. Dilute the matrix gel in serum-free cold cell culture medium (RPMI1640, EMEM, DMEM, etc.) from 5 mg/ml to 1 mg/ml. Place 100 µl of the diluted matrix gel into the upper chamber of a 24-well Transwell. Incubate the Transwell at 37 °C for at least 4 to 5 h for gelation. Treat each group of cells with 0.5 µM AZD9291. Wash the cells three times with cell culture medium containing 1% FBS (RPMI1640, EMEM, DMEM, etc.). Resuspend cells in the same medium at a density of 10^6 cells/ml. Gently wash the gelatinous matrix gel with warm serum-free cell culture medium. Place 100 µL of cell suspension on the matrix gel. Fill the lower chamber of the Transwell with 600 µL of cell culture medium containing 5 µg/ml fibronectin as a substrate for adhesion. Incubate at 37 °C for 20 to 24 h. Remove the Transwell from the 24-well plate and stain with Diff-Quick solution. Use a cotton swab to scrape off the non-invading cells from the top of the Transwell. Count the invading cells under an optical microscope. Please refer to the Supplemental Materials and Methods for detailed.

### Wound healing assay and analysis

Using a marker pen, draw evenly spaced horizontal lines on the back of a 6-well plate, approximately every 0.5 ~ 1 cm apart, crossing through each well, with at least 5 lines per well. Cells in logarithmic growth phase are digested with trypsin to obtain single-cell suspensions and seeded into the 6-well culture plate. Cells are plated at a density of 6w/cell per well, with the principle of achieving 100% confluence overnight. Treat each group of cells with 0.5 µM AZD9291. The next day, use a 200 µL pipette tip to mark the lid of the 6-well plate or a ruler parallel or perpendicular to the horizontal lines on the back, ensuring the pipette tip is vertical and not tilted. Remove the horizontal marker lines on the back of the 6-well plate. Take photographs under 4x magnification at 0, 12, and 24-hour time points for photography. Using Image J software (version 1.8; National Institutes of Health), open the images and randomly draw 6 to 8 horizontal lines, then calculate the mean distance between cells or the mean area of the marks. Experiment repeated thrice. Please refer to the Supplemental Materials and Methods for detailed.

### Annexin V/PI double staining assay [28, 29]

A total of 5 × 104 cells were seeded in 6‑well plates, cultured for 48 h at 37˚C and subsequently cells were collected. Cells were fixed with precooled 70% ethanol for 30 min at room temperature, and collected following centrifugation at 12,000 x g for 5 min at room temperature. Cells were fixed with precooled 70% ethanol for 30 min at room temperature and collected following centrifugation at 12,000 x g for 5 min at room temperature. Subsequently, 100 µl of Binding Buffer and 10 µl of FITC-labeled Annexin-V (20 µg/ml) were added, and the mixture was incubated at room temperature in the dark for 30 min. Then, 5 µl of PI (50 µg/ml) was added, and the reaction was incubated in the dark for an additional 5 min. Afterward, 400 µl of Binding Buffer was added, and flow cytometry analysis was immediately performed using FACScan (FCM, ThermoFisher Scientific, USA) and FlowJo 10.06 software (FlowJo LLC, Ashland, OR, USA) for quantitative detection of apoptotic cells. Specifically, Live cells cannot be stained by Annexin V-FITC or PI (lower left quadrant). Early apoptotic cells, which expose phosphatidylserine and have intact cell membranes, show positive staining with Annexin V-FITC and negative staining with PI (lower right quadrant). Dead or late apoptotic cells exhibit double-positive staining with Annexin V-FITC and PI (upper right quadrant). Please refer to the Supplemental Materials and Methods for detailed.

### Dual-luciferase reporter assay

Reporter plasmids containing Wild-type (WT) and mutant (MUT) circ_PPAPDC1A (3′UTR) or IGF1R (IGF1R-WT and IGF1R-MUT) (3′UTR) sequences were synthesized by Shanghai Gene Pharma Co., Ltd. The constructed reporter plasmids and miR-30a-3p mimic were co-transfected into HEK293T cells (National Collection of Authenticated Cells Cultures) using Lipofectamine® 3000 (Invitrogen; Thermo Fisher Scientific, Inc.). The luciferase activity was determined after 48-h transfection using a Dual-luciferase Assay System kit (cat. no. 16186; Thermo Fisher Scientific, Inc.). Firefly luciferase intensity was normalized to Renilla luciferase activity. Experiment repeated thrice. Please refer to the Supplemental Materials and Methods for detailed.

### Western-blotting

Cellular and tissues proteins were extracted using RIPA lysis buffer (cat no. 89900; Thermo Fisher Scientific, Inc.). Protein concentration was determined with a BCA Protein Assay kit (cat. no. 89-101-199; Thermo Fisher Scientific, Inc.), and 8 µg of protein was loaded per lane. PVDF membranes (cat. no.88585; Thermo Fisher Scientific, Inc.) were blocked with 5% BSA for 1 h at room temperature, then incubated overnight at 4 °C with primary antibodies (presented in Supplemental Table 9) and β-actin (1:1,000 dilution; cat. no. ab8245; Abcam Biotech Co., Ltd.). Afterward, membranes were incubated with secondary antibodies HRP-conjugated-goat anti-mouse IgG (H + L) (1:5,000 dilution; cat. no. ab205719, Abcam Biotech Co., Ltd.) at 37 °C. β-actin served as an internal control, and signaling was detected by ECL (Sigma-Aldrich, Inc.) and Band densitometry analysis was performed using ImageJ software (version 1.8; National Institutes of Health). Experiment repeated thrice. Please refer to the Supplemental Materials and Methods for detailed.

### Subcutaneous xenograft model

To validate the anti-tumor effect of circRNA on NSCLC cells, we used a subcutaneous xenograft model in nude mice. Cells were injected subcutaneously, and each group consisted of at least 5 mice. Tumor volume was monitored weekly using the formula V = 1/2×Width^2×Length. The growth curve was plotted based on length and time. After 5 weeks, the mice were euthanized, and their size and weight were measured. Subcutaneous tumor tissues were examined for histological changes, Ki-67 staining, and TUNEL assay. These analyses provided further insights into the impact of circRNAs on tumor growth and cell proliferation. The study was approved by the Animal Ethical and Welfare Committee of Xiangya Second Hospital, Central South University (approval No.2022127) in accordance with the Basel Declaration [30]. Please refer to the Supplemental Materials and Methods for detailed.

### Statistical analysis

The data are expressed as the mean ± standard deviation unless otherwise specified. Statistical analyses were performed using SPSS 18.0 software (SPSS Inc., Chicago, IL, USA) or GraphPad Prism 7 (GraphPad Software, Inc., La Jolla, CA, USA). Unpaired two-tailed Student’s t-test was applied to assess differences in cell viability, cell migration, invasion ability, cell apoptosis level, xenograft tumor volume, and xenograft tumor weight among various groups. The paired two-tailed Student’s t-test was employed to compare the expression of circ_PPAPDC1A/miR-30a-3p between OR tissues and their paired OS tissues. Spearman’s rho was utilized to evaluate the correlation between different variables. A significance level of *P* < 0.05 was considered statistically significant.

## Results

### circRNAs expression profiles

The total RNA of tumor tissues was extracted and Polyacrylamide gel electrophoresis revealed that the A260/280 ratios for all samples were between 1.90 and 2.08, while the A260/230 ratios ranged from 2.01 to 2.15 (Supplemental Table 2). We further labeled the extracted RNA, and the Dye concentrations were above 10.00 pmol/µL, the Specific Activity values were above 11 pmol/µL, and the total mass was above 15.00 µg (Supplemental Table 3). These results indicate that the extracted total RNA was of high purity and undegraded and high efficiency of RNA labeling, which is in accordance with the conditions for circRNAs microarray detection. circRNAs expression profiles were evaluated using a circRNAs chip assay. The circRNAs microarray hybridization signal diagram of the five tissue pairs collected from circRNAs chip scanner is presented in Supplemental Fig. 1A. Differential expression of circRNAs among samples was assessed using both the fold change (FC) threshold and filtering through Volcano Plot analysis. The circRNAs scatter plot (Supplemental Fig. 1C) and circRNAs volcano plot (Supplemental Fig. 1D) were generated to visualize statistically significant differences in expression between samples.

### circ_PPAPDC1A is significantly upregulated in Osimertinib resistance NSCLC tissues

**Fig. 1:**
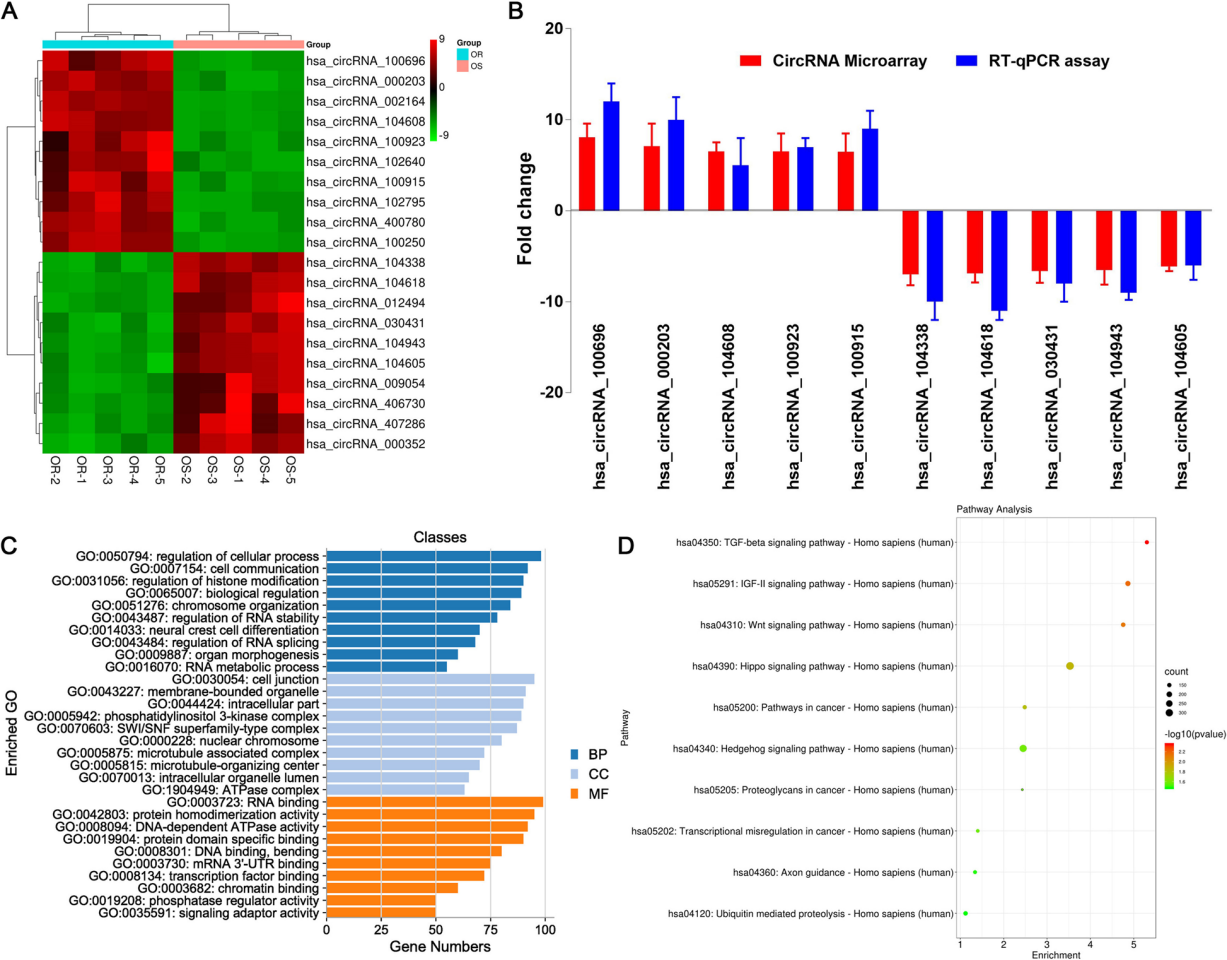
CircRNAs participated in the Osimertinib resistance. **A** Cluster heatmap of top 10 circRNAs; **B** RT-qPCR verification (*n* = 3): The RT-qPCR result is consistent with the circRNA chip result; **C** Gene Ontology annotation analysis. BP: Biological process, CC: Cellular component, MF: Molecular Function. **D** Kyoto Encyclopedia of Genes and Genomes (KEGG) signaling pathway Enrichment analysis. OS: Osimertinib Sensitive; OR: Osimertinib Resistance. RT-qPCR, reverse transcription-quantitative PCR. circ_100696: circ_PPAPDC1A

The circRNAs chip screening results unveiled a total of 468 differentially expressed circRNAs between Osimertinib-acquired resistant and sensitive tissues, meeting the criteria of Fold Change (FC) ≥ 2 and a significance level of *P* < 0.05. Among these, 242 circRNAs displayed an up-regulated expression pattern in resistant tissues, while 226 circRNAs showed down-regulation (Supplementary raw materials_ Differentially Expressed CircRNAs). Among the numerous differentially expressed circRNAs, we selected top ten most upregulated and downregulated circRNAs for clustering visualization analysis. The cluster heat map (Fig. 1A) revealed the top ten most upregulated and downregulated circRNAs and detailed annotation in Tables 1 and 2. circ_100696 was the most highly upregulated (8.08-fold) circRNAs in Osimertinib-resistant NSCLC tissues with highest raw intensity (Fig. 1A). Following literature analysis, 10 circRNAs were randomly selected for validation via RT-qPCR. They were split equally between up-regulated and down-regulated expression patterns. Selection criteria included over 6-fold expression differences and Raw Intensity above 100 in both sample chips. The results demonstrate that the RT-qPCR are consistent with the circRNAs microarray, validating the reliability of the circRNAs microarray results (Fig. 1B). Our study further conducted bioinformatics analyses, including Gene Ontology (GO) functional annotation analysis and KEGG signaling pathway analysis, on differentially expressed circRNAs coding genes. GO and KEGG analyses revealed that the differentially expressed circRNAs gene symbols were implicated in a range of biological processes (Fig. 1C, Supplemental Tables 4–6). Furthermore, they exhibited enrichment in signal pathways associated with tumorigenesis, notably the TGF-β pathway (Fig. 1D, Supplemental Table 7).

**Table 1:** Detailed annotation of the top 20 significantly upregulated circRNAs

circRNA	* P*-value	FC (abs)	source	chrom	type	GeneSymbol
hsa_circRNA_100696	0.031326071	8.080754	circBase	chr10	exonic	PPAPDC1A
hsa_circRNA_000203	0.002911971	7.082576	circBase	chr10	exonic	LARP4B
hsa_circRNA_002164	0.048545781	7.0777302	circBase	chr18	exonic	SS18
hsa_circRNA_104608	0.029909814	6.5289235	circBase	chr8	exonic	HOOK3
hsa_circRNA_100923	0.028496393	6.5087859	circBase	chr11	exonic	PICALM
hsa_circRNA_102640	0.032219657	6.5027121	circBase	chr2	exonic	ATAD2B
hsa_circRNA_100915	0.030601002	6.5012908	circBase	chr11	exonic	PICALM
hsa_circRNA_102795	0.007949059	5.9894076	circBase	chr2	exonic	UXS1
hsa_circRNA_400780	0.025332973	5.7282039	25,242,744	chr11	exonic	LRP5
hsa_circRNA_100250	0.041990494	5.7205296	circBase	chr1	exonic	ROR1
hsa_circRNA_103031	0.008421471	5.7140434	circBase	chr20	exonic	ITCH
hsa_circRNA_102358	0.02379793	5.6880425	circBase	chr18	exonic	HAUS1
hsa_circRNA_075650	0.032940532	5.6874403	circBase	chr6	exonic	RANBP9
hsa_circRNA_090183	0.012231177	5.6814072	circBase	chrX	exonic	PRRG1
hsa_circRNA_100926	0.046069869	5.653654	circBase	chr11	exonic	PICALM
hsa_circRNA_403893	0.018746645	5.6513101	25,242,744	chr7	exonic	KMT2E
hsa_circRNA_012872	0.047499759	5.6290967	circBase	chr1	exonic	JAK1
hsa_circRNA_102157	0.048647835	5.6233631	circBase	chr17	exonic	TLK2
hsa_circRNA_404667	0.044082256	5.6027006	25,070,500	chr1	exonic	WDR26
hsa_circRNA_001520	0.046621155	5.6008463	circBase	chr5	sense overlapping	DCP2

**Table 2:** Detailed annotation of the top 20 significantly downregulated circRNAs

circRNA	* P*-value	FC (abs)	source	chrom	type	GeneSymbol
hsa_circRNA_104338	0.041765932	6.9966654	circBase	chr7	exonic	CREB5
hsa_circRNA_104618	0.04671275	6.8901572	circBase	chr8	exonic	PRKDC
hsa_circRNA_012494	0.048150871	6.8101532	circBase	chr1	exonic	ZFYVE9
hsa_circRNA_030431	0.0231521	6.6169877	circBase	chr13	exonic	TBC1D4
hsa_circRNA_104943	0.042411323	6.5242817	circBase	chr9	exonic	NUP214
hsa_circRNA_104605	0.044096564	6.137549	circBase	chr8	exonic	RNF170
hsa_circRNA_009054	0.039487895	5.9959357	circBase	chr5	exonic	MCC
hsa_circRNA_406730	0.02724178	5.9649974	25,070,500	chr6	sense overlapping	LOC285768
hsa_circRNA_407286	0.043347887	5.8578241	25,070,500	chrX	sense overlapping	SHROOM4
hsa_circRNA_000352	0.014094106	5.846358	circBase	chr11	antisense	BIRC3
hsa_circRNA_043598	0.005301978	5.8279019	circBase	chr17	exonic	KRT19
hsa_circRNA_103060	0.023233395	5.7592248	circBase	chr20	exonic	RPN2
hsa_circRNA_102211	0.023255909	5.6872669	circBase	chr17	exonic	PGS1
hsa_circRNA_039219	0.019539192	5.6856951	circBase	chr16	exonic	GPT2
hsa_circRNA_101857	0.042366854	5.6748218	circBase	chr16	exonic	PDPR
hsa_circRNA_102863	0.016844696	5.6744773	circBase	chr2	exonic	TTN-AS1
hsa_circRNA_406705	0.002954174	5.6549713	25,070,500	chr5	sense overlapping	TTC1
hsa_circRNA_007912	0.000840415	5.6543105	circBase	chr3	exonic	ARMC8
hsa_circRNA_104004	0.044938591	5.6486745	circBase	chr5	exonic	SLIT3
hsa_circRNA_405386	0.013243433	5.6478525	25,070,500	chr15	intronic	PARP6

#### circ_PPAPDC1A is significantly upregulated in Osimertinib resistance NSCLC cells

**Fig. 2:**
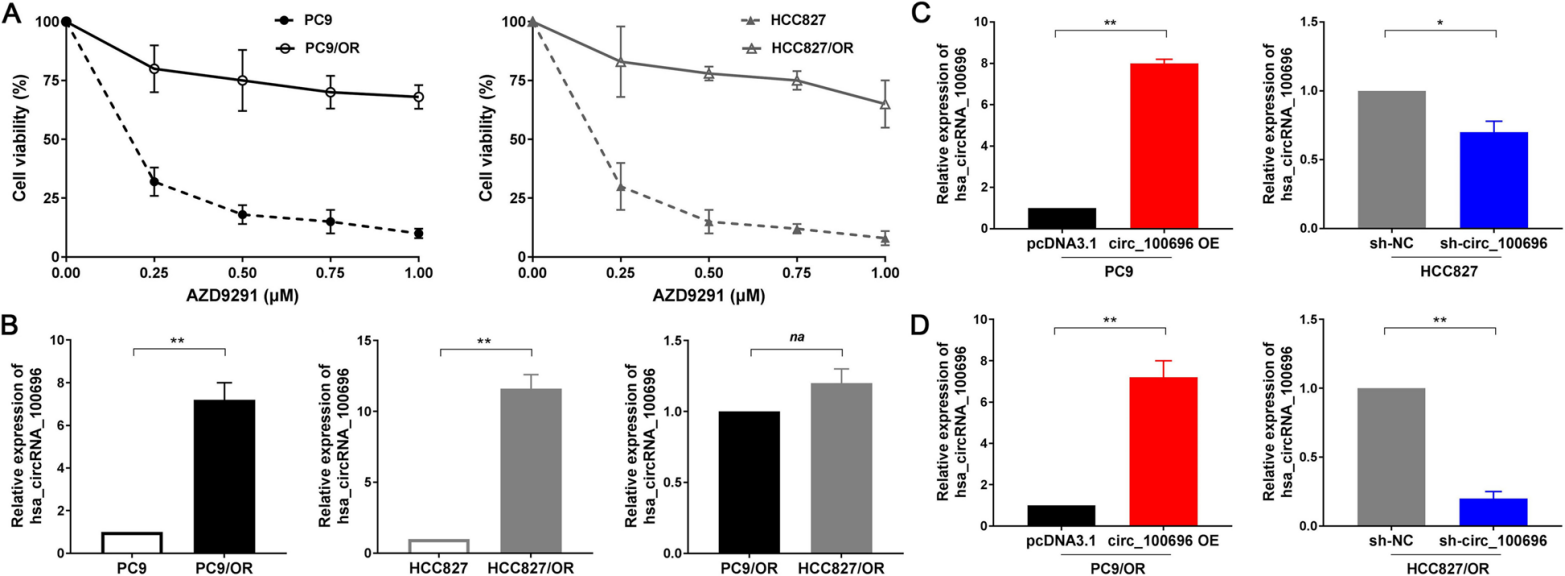
circ_PPAPDC1A expression is elevated in Osimertinib resistance NSCLC tissues and cells. **A** Successful establishment of Osimertinib resistance cells, named as PC9/OR and HCC827/OR. The transfection efficiency was assessed by RT-qPCR. **B** RT-qPCR analysis of circ_100696 expression in PC9/OR and HCC827/OR cells. circRNA_100696 expression levels were normalized to GAPDH. **C**, **D** Successful establishment of circRNA_100696 overexpression and knockdown cellular models. circ_100696 overexpression and knockdown lentiviral vectors were subsequently stably transfected them into PC9/OR and HCC827/OR cells, as well as their parental PC9 and HCC827 cells. *n* = 3. *P < 0.05, **P < 0.01, na: no statistical significance. Osimertinib Sensitive; OR: Osimertinib Resistance. RT-qPCR, reverse transcription-quantitative PCR. circ_100696: circ_PPAPDC1A

Upon querying the circBase database (http://circrna.org/), circRNA_100696 was identified to be situated on chromosome 10 at position q26.12:122273422–122,280,607. It represents the most prevalent circRNAs derived from exons and is linked to the gene symbol PPAPDC1A (NM_001030059), so, circ_100696 is also referred to as circ_PPAPDC1A. This circRNAs is formed by the splicing of the 3rd, 4th, and 5th exons of the PPAPDC1A gene. The genomic length is 7185 bp, while the spliced sequence length is 280 bp (Supplemental Fig. 2A, Supplemental Table 8). In our study, we selected Osimertinib-sensitive PC9 and HCC827 cell lines as parental cells. Highly Osimertinib-resistant cell lines were established through prolonged drug exposure (gradually increasing AZD9291 concentration from 10nM to 500nM) and extended screening (maintaining resistance after drug withdrawal), and specifically referred to as PC9/OR and HCC827/OR. CCK-8 validation assays revealed that AZD9291 exhibited significant dose-dependent proliferation inhibition in non-small cell lung cancer PC9 and HCC827 cells, with IC50 values of 0.17µM and 0.15µM, respectively. However, at concentrations of 0.75-1µM, AZD9291 only achieved around 25% proliferation inhibition in PC9/OR and HCC827/OR cells, indicating high resistance in these cell lines (Fig. 2A). We conducted fluorescence in situ hybridization (FISH) experiments to determine the subcellular localization of circRNA_100696 expression. The results revealed that circRNA_100696 is widely present in PC9/OR and HCC827/OR cells, with the primary localization being enriched in the cytoplasm (Supplemental Fig. 2B). Also, RT-qPCR assay indicate that circ_PPAPDC1A exhibits significantly lower expression in human normal lung epithelial cells EAS-2B compared to PC9 and HCC827 cells (*P* < 0.05; Supplemental Fig. 3A). Meanwhile, RT-qPCR assay showed that circRNA_100696 expression is significantly higher in PC9/OR and HCC827/OR cells compared to the parental PC9 and HCC827 cells (*P* < 0.05; Fig. 2B). Additionally, we observed a slightly lower expression of circRNA_100696 in PC9/OR cells compared to HCC827/OR cells, although this difference is not statistically significant (*P* > 0.05; Fig. 2B). Furthermore, we constructed overexpression and knockdown lentiviral vectors for circRNA_100696, and subsequently stably transfected them into PC9/OR and HCC827/OR cells, as well as their parental cells, and circRNA_100696 overexpression and knockdown cellular models were successfully constructed (*P* < 0.05; Fig. 2C, D).

### circ_PPAPDC1A decreased Osimertinib sensitivity of NSCLC and suppresses the cell malignant behaviors in vitro

**Fig. 3:**
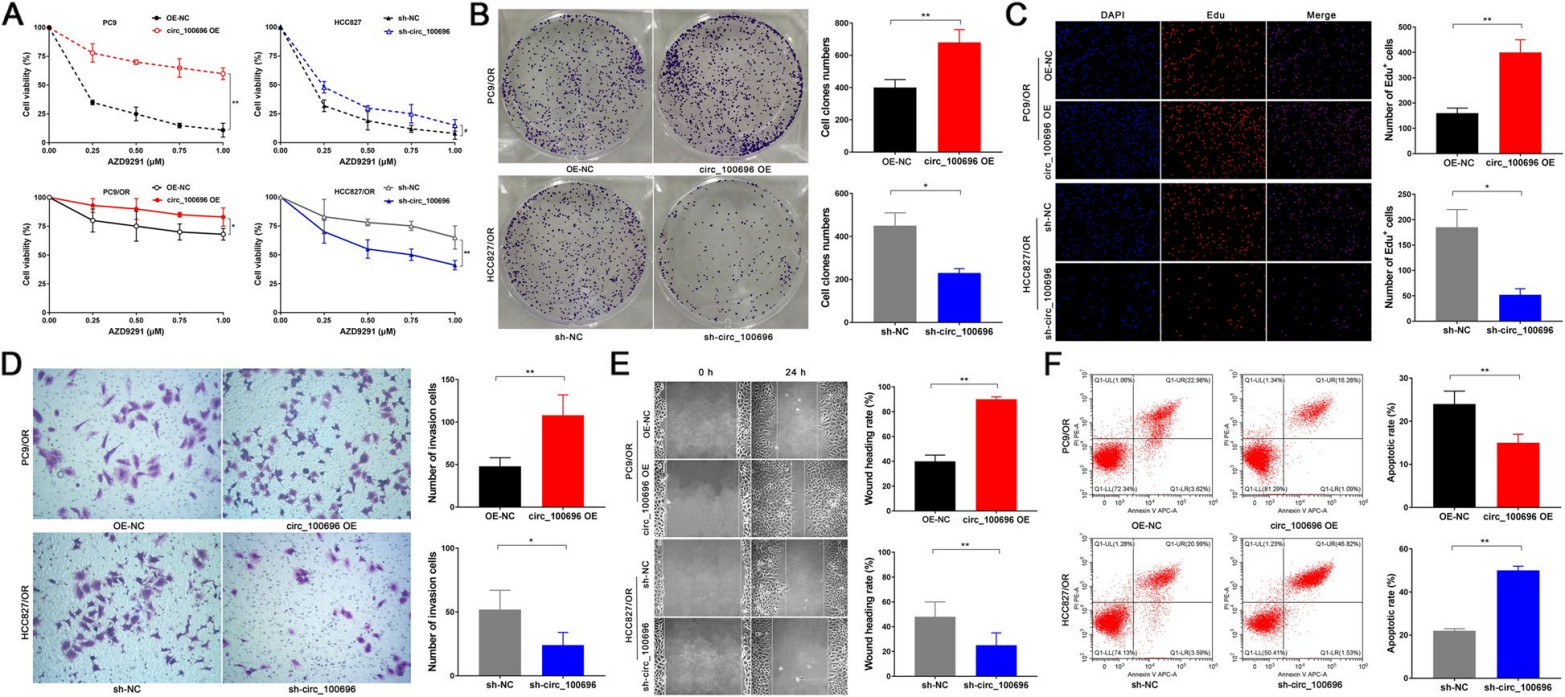
circ_PPAPDC1A decreased Osimertinib sensitivity of NSCLC and suppresses the cell malignant behaviors in vitro. **A** Cell counting kit-8 (CCK8) assay was conducted to determine the IC50 value of Osimertinib. **B** Soft agar colony assay was employed to assess colony formation ability. **C** EdU assay was performed to evaluate DNA synthesis ability. **D** Transwell chamber assay was performed to analyze cell invasion ability. **D** Cell scratch assay was carried out to examine Cell migration ability. **F** and Annexin V-FITC/PI assay was performed to investigate cell apoptosis. *n* = 3. *P < 0.05, **P < 0.01. Osimertinib Sensitive; OR: Osimertinib Resistance. RT-qPCR, reverse transcription-quantitative PCR. circ_100696: circ_PPAPDC1A

To explore the effect of circ_PPAPDC1A on cell proliferation and Osimertinib sensitivity in NSCLC cells, we exposed PC9, HCC829, PC9/OR, and HCC829/OR cells to varying concentrations of AZD9291 (0 µM, 0.25 µM, 0.5 µM, 0.75 µM, 1.0 µM) in vitro. CCK-8 assay showed that circ_PPAPDC1A has no significant effect on the cell proliferation of PC9 and HCC827 cells (*P* < 0.05; Supplemental Fig. 3B). But, CCK-8 assay revealed that circ_PPAPDC1A overexpression significantly reduces the sensitivity of PC9 and PC9/OR cells to AZD9291; conversely, circ_PPAPDC1A interference markedly enhances AZD9291 sensitivity in HCC827/OR cells, partially reversing resistance (*P* < 0.05; Fig. 3A). However, interfering with the expression of circ_PPAPDC1A in HCC827 cells had no significant effect on the sensitivity to AZD9291. Thus, we focused our investigation on PC9/OR and HCC829/OR cells. These cell lines were treated with 0.5µM AZD9291 for 14 days in vitro. Subsequent assays, including the soft agar colony assay (*P* < 0.05; Fig. 3B), EdU assay (*P* < 0.05; Fig. 3C), Transwell chamber assay (*P* < 0.05; Fig. 3D), cell scratch assay (*P* < 0.05; Fig. 3E) and Annexin V-FITC/PI assay (*P* < 0.05; Fig. 3F) demonstrated that overexpression of circ_PPAPDC1A contribute to heightened clonogenicity and enhanced DNA replication in PC9/OR cells, facilitating cellular invasion and migration while restraining apoptosis. In contrast, interference with circ_PPAPDC1A leads to decreased clonogenicity and hindered DNA replication in HCC827/OR cells, effectively reducing cellular invasion and migration, while promoting apoptosis.

### circ_PPAPDC1A serves as an Efficient miRNA Sponge for miR-30a-3p

**Fig. 4:**
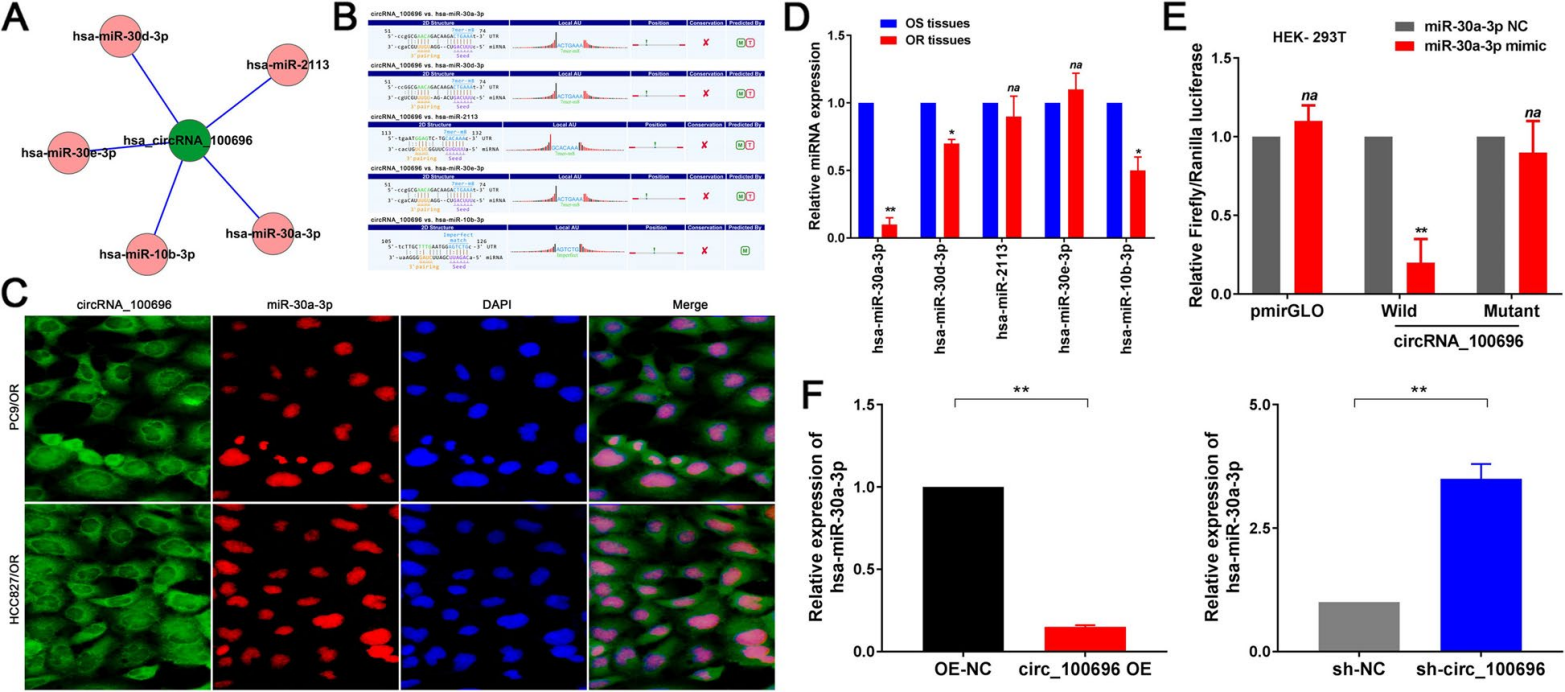
circ_PPAPDC1A serves as an Efficient miRNA Sponge for miR-30a-3p in Osimertinib resistance NSCLC cell. **A** The top five targeted miRNAs by circ_PPAPDC1A. **B** circ_100696-miRNA interactions were predicted, and the putative binding sites between circ_100696 and top five targeted miRNAs are shown. **C** RT-qPCR analysis demonstrated that, hsa-miR-30a-3p, hsa-miR-30d-3p, and hsa-miR-10b-3p were downregulated in the Osimertinib resistance tissues, while hsa-miR-30a-3p exhibited the most pronounced downregulation. **D** Fluorescence in situ hybridization (FISH) found that circ_100696 and miR-30a-3p were located in the cytoplasm and nucleus respectively. **E** Dual-luciferase reporter assay confirmed that miR-30a-3p could competitively targeted circ_100696 (HEK-293T). **F** RT-qPCR assay demonstrated that miR-30a-3p expression was significantly decreased in circ_100696 OE group and increased in sh-circ_100696 group compared with control group. *n* = 3. *P < 0.05, **P < 0.01, na: no statistical significance. Osimertinib Sensitive; OR: Osimertinib Resistance. RT-qPCR, reverse transcription-quantitative PCR. circ_100696: circ_PPAPDC1A. OE: OverExpression

CircRNAs are known to function as miRNA sponges, regulating the expression of specific target genes. In our study, we identified hsa-miR-30a-3p, hsa-miR-30d-3p, hsa-miR-2113, hsa-miR-30e-3p, and hsa-miR-10b-3p as the top five targeted miRNAs by circ_PPAPDC1A (Fig. 4A). Utilizing Arraystar’s exclusive software, we established that these miRNAs exhibited specific binding affinity with circ_PPAPDC1A (*P* < 0.05; Fig. 4B). Subsequent RT-qPCR analysis demonstrated that, compared to the corresponding Osimertinib sensitive tissues, hsa-miR-30a-3p, hsa-miR-30d-3p, and hsa-miR-10b-3p were downregulated in the Osimertinib resistance tissues, while hsa-miR-2113 and hsa-miR-30e-3p showed no significant expression differences. Notably, among these miRNAs, hsa-miR-30a-3p exhibited the most pronounced downregulation (*P* < 0.05; Fig. 4D). Pearson’s correlation analysis highlighted a negative correlation trend between circ_PPAPDC1A and miR-30a-3p expression in Osimertinib resistance tissues, although there was no statistical significance due to the small sample size. (*n* = 5; *r*=-0.81, *P* = 0.11; Supplemental Fig. 4A). Thus, miR-30a-3p was selected as a potential target miRNA for further investigation. To validate the interaction, a Dual-luciferase reporter assay (Table 3) was conducted. In HEK-293T cells co-transfected with pmirGLO-circ_100696-wt and miR-30a-3p mimic, luciferase activity notably decreased compared to cells co-transfected with pmirGLO- circ_100696-wt and miR-30a-3p NC (*P* < 0.05; Fig. 4E). However, no significant alteration in luciferase activity was observed in cells co-transfected with pmirGLO-circ_100696-mut and miR-30a-3p mimic or pmirGLO-circ_100696-mut and miR-30a-3p NC (*P* > 0.05; Fig. 4E). To further validate the functional targeting of miR-30a-3p by circ_PPAPDC1A, we employed RT-qPCR. This analysis demonstrated a significant decrease in miR-30a-3p expression in the circ_100696 OV group and an increase in the sh-circ_100696 group compared to the OV-NC and sh-NC groups (*P* < 0.05; Fig. 4F). Fluorescence in situ hybridization (FISH) analysis further confirmed the localization of circ_PPAPDC1A and miR-30a-3p in the cytoplasm and nucleus, respectively (Fig. 4D). These findings collectively indicate that miR-30a-3p specifically binds to circ_PPAPDC1A, and circ_PPAPDC1A negatively regulates miR-30a-3p expression in Osimertinib-resistant cells, suggesting that miR-30a-3p is one of the functional target miRNAs of circ_PPAPDC1A, exerting a miRNA sponge effect.

**Table 3:** Construction of circRNA-wild-type/mutant plasmids

Plasmids	Sequences
circ_100696-3’UTR-wild type (wt)	GCAATTTCTTTCCTCACACCCCTGGCTGTTATTTGTGTGGTGAAAATTATCCG**GC**G**AACA**GACAA**GACTGAAA**TTAAGGAAGCCTTCTTAGCGGTGTCCTTGGCTCTTGCTTTGAATGGAGTCTGCACAAACACTATTAAATTAATAGTGGGAAGACCTCGCCCCGATTTCTTTTACCGCTGCTTTCCAGATGGAGTGATGAACTCGGAAATGCATTGCACAGGTGACCCCGATCTGGTGTCCGAGGGCCGCAAAAGCTTCCCCAGCATCCATTCCTCCT
circ_100696-3’UTR-mutation (mut)	GCAATTTCTTTCCTCACACCCCTGGCTGTTATTTGTGTGGTGAAAATTATCCG**GC**G**AACA**GACAA**GTGACTTTTT**AAGGAAGCCTTCTTAGCGGTGTCCTTGGCTCTTGCTTTGAATGGAGTCTGCACAAACACTATTAAATTAATAGTGGGAAGACCTCGCCCCGATTTCTTTTACCGCTGCTTTCCAGATGGAGTGATGAACTCGGAAATGCATTGCACAGGTGACCCCGATCTGGTGTCCGAGGGCCGCAAAAGCTTCCCCAGCATCCATTCCTCCT

### miR-30a-3p plays a tumor suppressor role in Osimertinib resistance

**Fig. 5:**
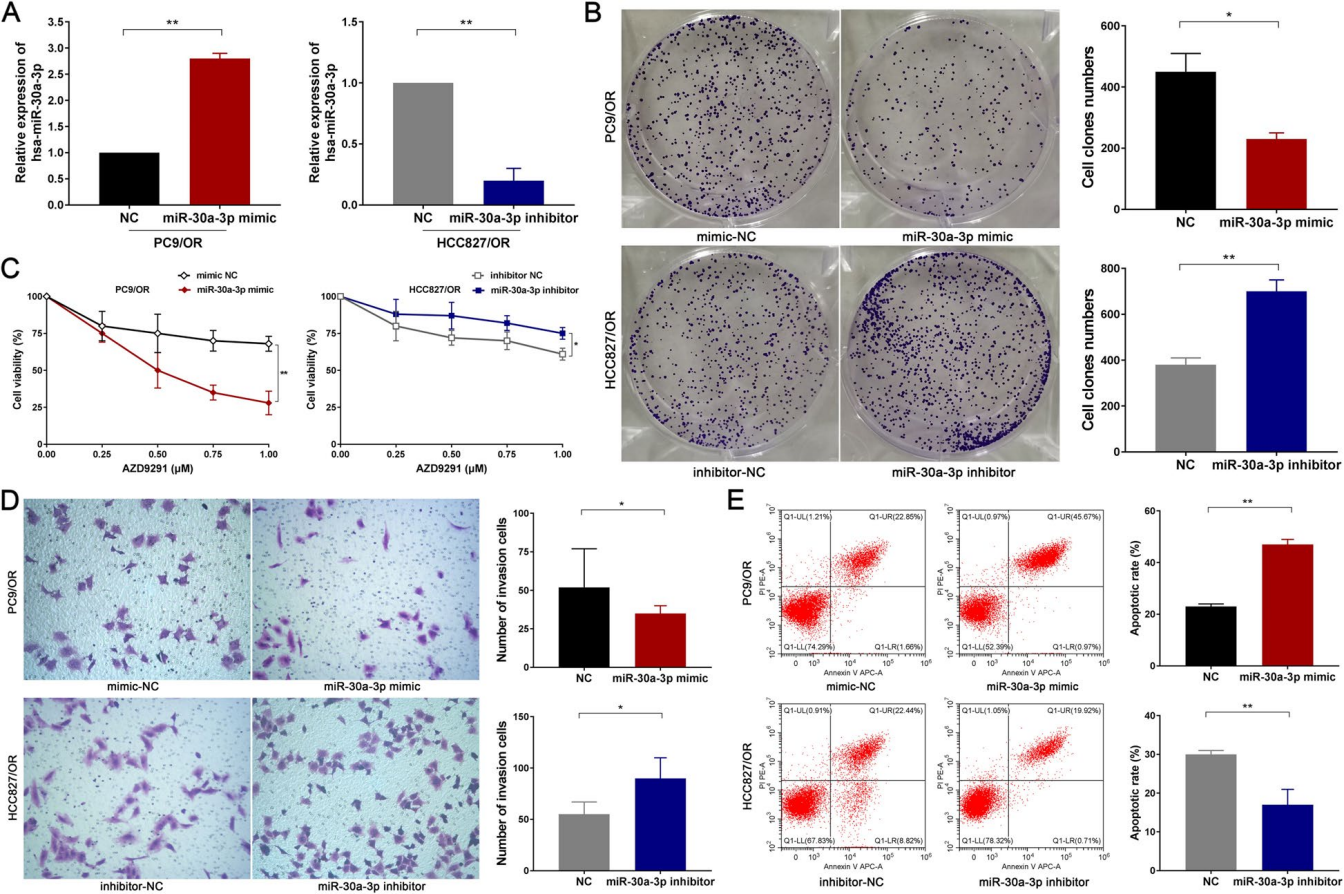
miR-30a-3p plays a tumor suppressor role in NSCLC with Osimertinib resistance. **A** Successful establishment of miR-30a-3p overexpression and knockdown cellular models: miR-30a-3p mimic and inhibitor and subsequently stably transfected them into PC9/OR and HCC827/OR cells, respectively. The transfection efficiency was assessed by RT-qPCR. **B** Cell counting kit-8 (CCK8) assay was conducted to determine the IC50 value of Osimertinib. **C** Soft agar colony assay was employed to assess colony formation ability. **D** Transwell chamber assay was performed to analyze cell invasion ability. **F** and Annexin V-FITC/PI assay was performed to investigate cell apoptosis. *P < 0.05, **P < 0.01. Osimertinib Sensitive; OR: Osimertinib Resistance. circ_100696: circ_PPAPDC1A

To explore the effect of miR-30a-3p on Osimertinib sensitivity in NSCLC cells, we constructed miR-30a-3p mimic and inhibitor and subsequently stably transfected them into PC9/OR and HCC827/OR cells through Lipofectamine ^TM3000^ (*P* < 0.05; Fig. 5A). After transfection, we exposed PC9/OR and HCC827/OR cells to varying concentrations of AZD9291 (0 µM, 0.25 µM, 0.5 µM, 0.75 µM, 1.0 µM). The CCK-8 assay revealed that the miR-30a-3p mimic significantly heightens the responsiveness of PC9/OR cells to AZD9291, partially reversing resistance. Conversely, the miR-30a-3p inhibitor notably diminishes AZD9291 sensitivity in HCC827/OR cells (*P* < 0.05; Fig. 5B). Furthermore, we subjected PC9/OR and HCC827/OR cells to 0.5 µM AZD9291 treatment for 14 days in vitro. Subsequent analyses, encompassing the soft agar colony assay (*P* < 0.05; Fig. 5C), Transwell chamber assay (*P* < 0.05; Fig. 5D), and Annexin V-FITC/PI assay (*P* < 0.05; Fig. 5E) illustrated that upregulation of miR-30a-3p expression curbs the clonogenic and invasive capabilities of PC9/OR cells. In contrast, downregulation of miR-30a-3p expression amplifies the clonogenic and invasive tendencies of HCC827/OR cells. Additionally, augmenting miR-30a-3p expression fosters apoptosis in PC9/OR cells, whereas suppressing miR-30a-3p expression impedes apoptosis in HCC827/OR cells.

### IGF1R identified as a functional target gene for miR-30a-3p

**Fig. 6:**
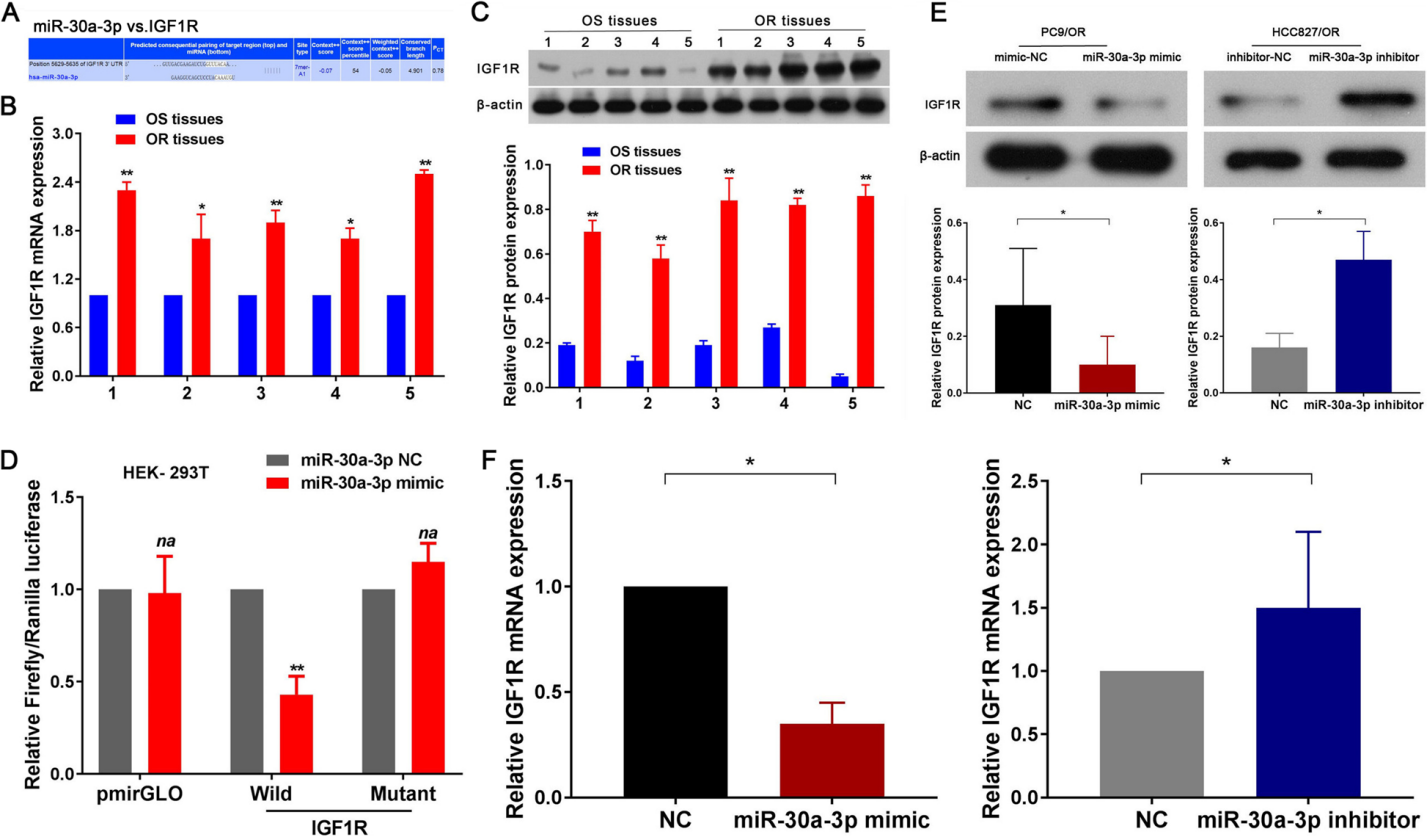
IGF1R identified as a functional target gene for miR-30a-3p in Osimertinib resistance NSCLC cell. **A** Schematic representation showing that miR-370-3p specifically binds to IGF1R. RT-qPCR (**B**) and Western-blot (**C**) assay were confirmed that IGF1R mRNA and protein expression were significantly upregulated in Osimertinib resistance NSCLC tissues. **D** Dual-luciferase reporter assay confirmed that miR-30a-3p could competitively targeted wt 3’UTR sequence of IGF1R (HEK-293T). RT-qPCR (**E**) and Western-blot (**F**) assay demonstrated that IGF1R mRNA and protein expression were significantly decreased in miR-30a-3p mimic and increased in miR-30a-3p inhibitor group compared with control group. *n* = 3. *P < 0.05, **P < 0.01, na: no statistical significance. Osimertinib Sensitive; OR: Osimertinib Resistance. RT-qPCR, reverse transcription-quantitative PCR. circ_100696: circ_PPAPDC1A. UTR, untranslated region. Wt, wild type

The potential targets of miR-30a-3p were predicted through three miRNA target gene databases (miRWalk, TargetScan, and miRanda), which collectively listed 315 genes. Among these genes, IGF1R, a well-known tumor suppressor, exhibited a high prediction score (as shown in Fig. 6A). Further investigation using RT-qPCR and western blot assays revealed a significant upregulation of IGF1R mRNA and protein in Osimertinib-resistant tissues compared to their Osimertinib-sensitive counterparts (*P* < 0.05; Fig. 5B, C). Pearson’s correlation analysis highlighted a negative correlation trend between IGF1R mRNA and miR-30a-3p expression in Osimertinib resistance tissues, although there was no statistical significance due to the small sample size (*n* = 5; *r*=-0.74, *P* = 0.15; Supplemental Fig. 4B). Based on microRNA Response Element (MRE) analysis, miR-30a-3p was found to have potential binding sites within the 3’-UTR region of IGF1R. Therefore, we inserted both the wild-type (wt) and mutated IGF1R-3’-UTR sequences, each containing the predicted binding site, into luciferase reporter plasmids. Subsequent luciferase assays revealed that the relative luciferase activity in the IGF1R-3’-UTR-wt and miR-30a-3p mimic co-transfection group was significantly decreased compared to the miR-30a-3p negative control (NC) or IGF1R-3’-UTR-mut groups (both *P* < 0.05; Fig. 5D). Furthermore, we investigated the downstream effects of miR-30a-3p on IGF1R. Our RT-qPCR and western blot assays demonstrated that IGF1R mRNA and protein levels were significantly decreased in the miR-30a-3p mimic group and significantly increased in the miR-30a-3p inhibitor group compared to the miR-30a-3p NC group (both *P* < 0.05; Fig. 5E, F). These findings identified that IGF1R was a functional target gene for miR-30a-3p.

Studies have reported that hat LINC01436 promotes NSCLC progression by acting as a microRNA (miR)-30a-3p sponge to regulate the expression of its target gene EPAS1. There is a possibility that EPAS1 might be involved in Osimertinib resistance. Therefore, our study additionally explored the role of EPAS1 in Osimertinib resistance. However, our supplementary studies found that there is no significant difference in EPAS1 mRNA and protein expression between NSCLC Osimertinib sensitive and resistance cells (both *P* > 0.05; Supplemental Fig. 5A, B). Additionally, we knocked out EPAS1 to explore its impact on Osimertinib sensitivity in NSCLC cells. Subsequently, PC9, HCC829, PC9/OR, and HCC829/OR cells were exposed to various concentrations of AZD9291 (0 µM, 0.25 µM, 0.5 µM, 0.75 µM, 1.0 µM) in vitro. The CCK-8 assay results showed that knocking out EPAS1 does not affect the sensitivity of PC9, HCC829, PC9/OR, and HCC829/OR cells to Osimertinib (both *P* > 0.05; Supplemental Fig. 5C, D). Hence, our supplementary experiments suggest that while EPAS1 may play a role in the malignant progression of NSCLC, its involvement in the development of Osimertinib resistance may be minimal.

### circ_PPAPDC1A promotes Osimertinib resistance by regulating miR-30a-3p expression and activating IGF1R/PI3K/ AKT/mTOR pathway

**Fig. 7:**
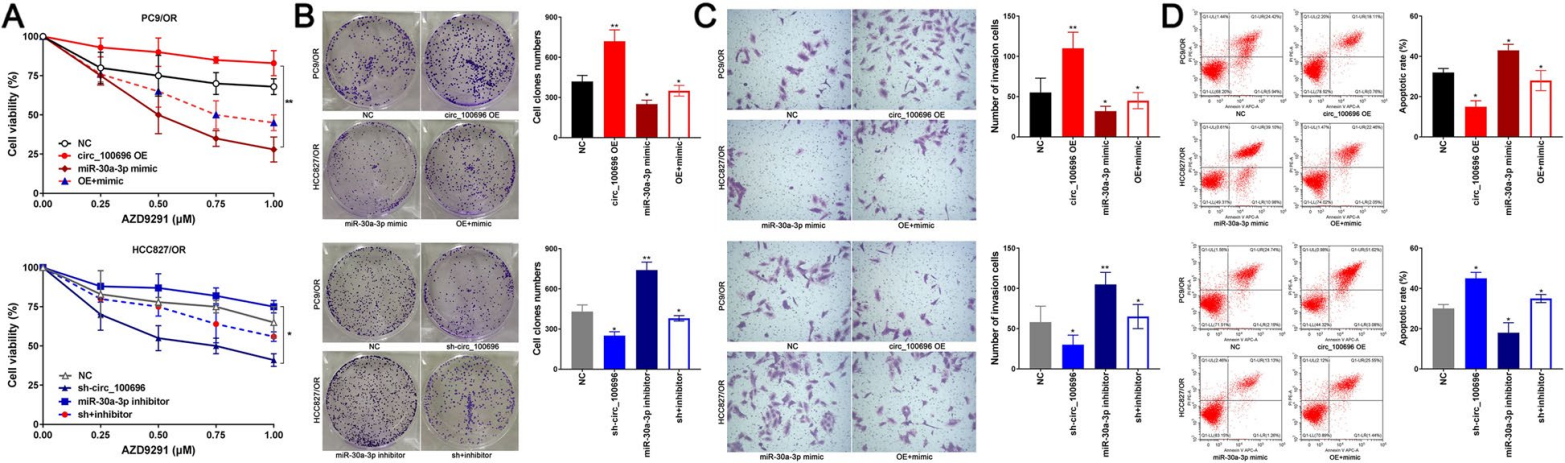
circ_PPAPDC1A promotes Osimertinib resistance in NSCLC by regulating the expression of miR-30a-3p. PC9/OR cells were co-transfected with the circ_100696 OE vector and miR-30a-3p mimics. HCC827/OR cells were also co-transfected with sh-circ_100696 and miR-30a-3p inhibitors. **A** Cell counting kit-8 (CCK8) assay (**B**) Soft agar colony assay (**C**) Matrigel invasion and **D** Annexin V-FITC/PI assay showed the effects of circ_PPAPDC1A/miR-30a-3p axis on cell proliferation, clone formation invasion and apoptosis. circ_PPAPDC1A promoted cell proliferation and invasion while inhibiting cell apoptosis. But these effects were reversed following treatment with miR-30a-3p mimic and mimic inhibitor. *n* = 3. *P < 0.05, **P < 0.01, na: no statistical significance. Osimertinib Sensitive; OR: Osimertinib Resistance. RT-qPCR, reverse transcription-quantitative PCR. circ_100696: circ_PPAPDC1A. OE: OverExpression

**Fig. 8:**
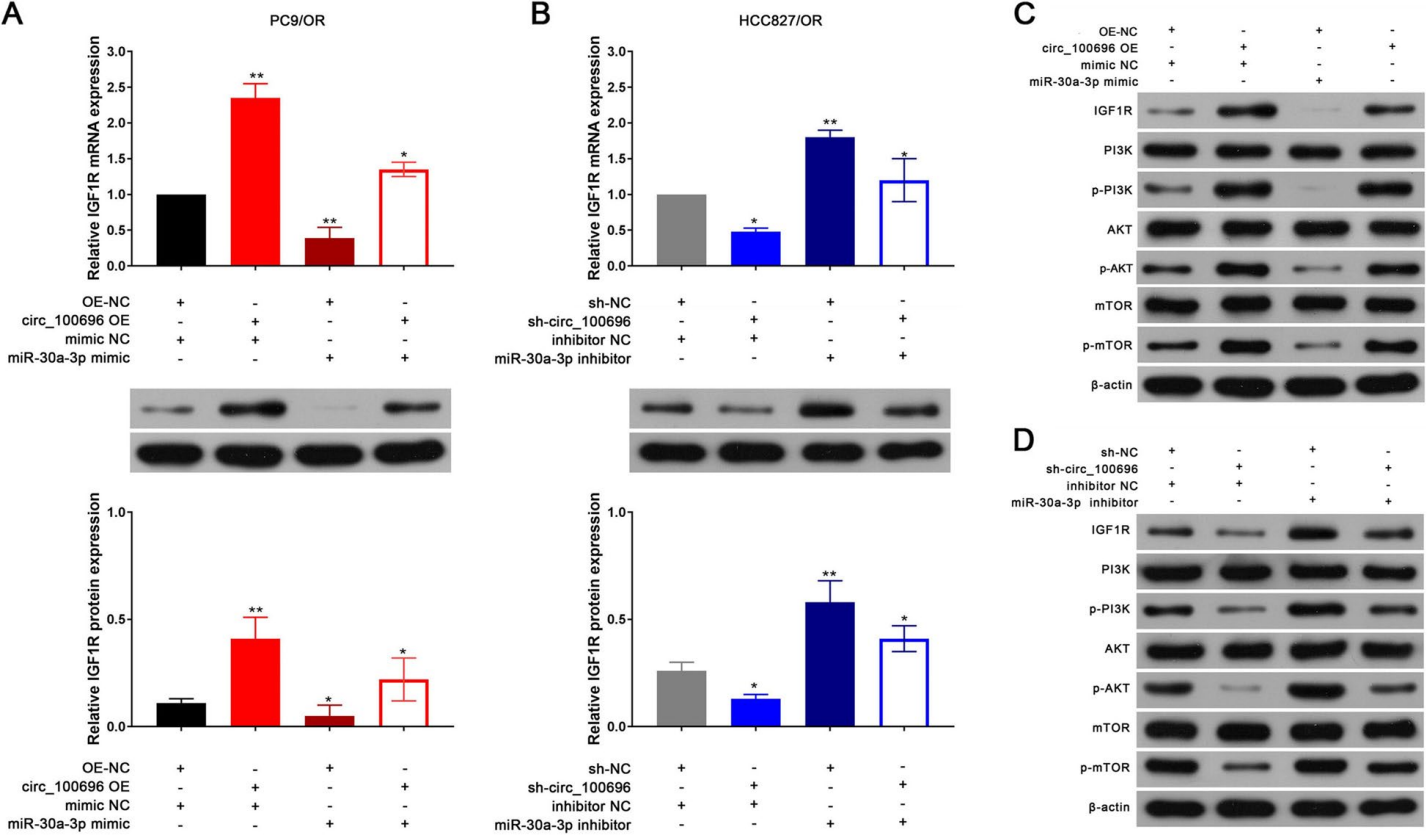
IGF1R/PI3K/AKT/mTOR pathway can activated by the circ_PPAPDC1A /miR-30a-3p axis. PC9/OR cells were co-transfected with the circ_100696 OE vector and miR-30a-3p mimics. HCC827/OR cells were also co-transfected with sh-circ_100696 and miR-30a-3p inhibitors. RT-qPCR and Western-blot assay analysis of IGF1R mRNA and protein expression (**A**-**B**) and (**B**) PI3K, p-PI3K, AKT, p-AKT, mTOR, p-mTOR protein expression in circ_100696 and miR-30a-3p transfection groups. circ_PPAPDC1A can activate IGF1R/PI3K/AKT/mTOR pathway by regulating the expression of miR-30a-3p, but these effects can be reversed by miR-30a-3p. *n* = 3. *P < 0.05, **P < 0.01, na: no statistical significance. Osimertinib Sensitive; OR: Osimertinib Resistance. RT-qPCR, reverse transcription-quantitative PCR. circ_100696: circ_PPAPDC1A. OE: OverExpression

Several rescue experiments were conducted to validate the role of the circ_PPAPDC1A/miR-30a-3p/IGF1R axis in regulating Osimertinib resistance of NSCLC. Results from CCK-8, Soft agar colony, Matrigel invasion, and Annexin V-FITC/PI assays demonstrated that overexpression of circ_PPAPDC1A promoted cell proliferation and invasion while inhibiting cell apoptosis. Conversely, silencing circ_PPAPDC1A inhibited cell proliferation and invasion, promoting cell apoptosis. Notably, these effects were reversed following treatment with miR-30a-3p mimic and mimic inhibitor (*P* < 0.05; Fig. 7A-C). We also assessed the levels of IGF1R expression by RT-qPCR and Western blot assays. The results revealed that IGF1R mRNA and protein expression significantly increased in the circ_PPAPDC1A overexpression group and decreased in the sh-circ_PPAPDC1A group. However, these effects were mitigated by miR-30a-3p mimic and inhibitor, respectively (*P* < 0.05; Fig. 8A, B). Furthermore, our research emphasized the downstream PI3K/AKT/ mTOR signaling pathway of IGF1R gene. Western blot assay results indicated that the protein expression levels of p-PI3K, p-AKT, and p-mTOR significantly decreased in circ_PPAPDC1A overexpression groups and increased in sh-circ_PPAPDC1A groups. However, these changes did not affect the expression of PI3K, AKT, and mTOR proteins. These findings suggest that circ_PPAPDC1A can activate the PI3K/ AKT/mTOR signaling pathway through IGF1R, while inhibition has the opposite effect. Importantly, these effects were again reversed following treatment with miR-30a-3p mimic and mimic inhibitor (*P* < 0.05; Fig. 8C, Supplemental Tables 9–11). In summary, circ_PPAPDC1A promotes Osimertinib resistance by regulating miR-30a-3p expression and activating IGF1R/PI3K/AKT/mTOR pathway.

### circ_PPAPDC1A suppresses Osimertinib sensitivity of NSCLC in vivo

**Fig. 9:**
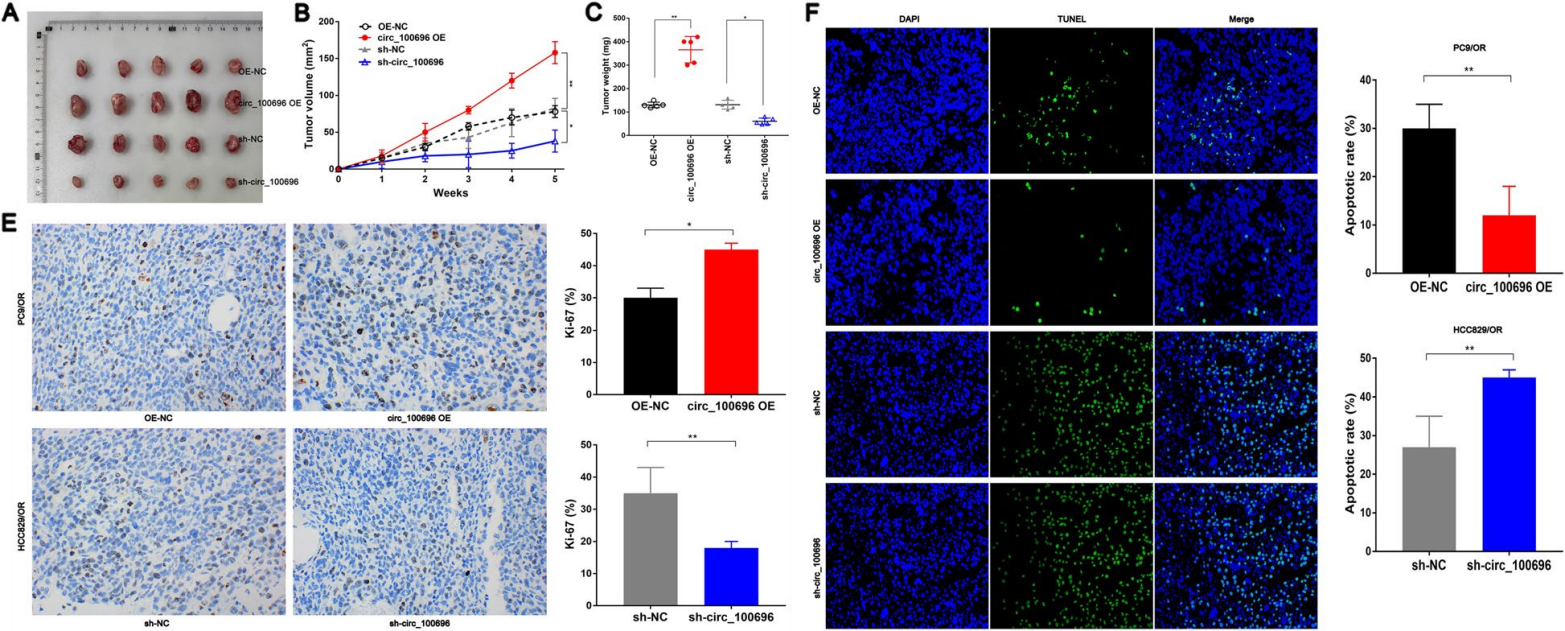
circ_PPAPDC1A suppresses Osimertinib sensitivity of NSCLC in vivo. **A** We established subcutaneous tumor models in nude mice via PC9/OR and HCC827/OR cells subcutaneous injection with AZD9291 treatment. After 5 weeks of feeding, the mice were euthanized, and nude mice were taken out to measure their size and weighed. **B** Xenograft tumor growth curves and **C** Xenograft tumor weight revealed that circ_PPAPDC1A suppresses Osimertinib sensitivity of NSCLC in in vivo. **D** The percentage of Ki67-positive cells in xenograft tumors was measured. **E** TUNEL assay was performed to determine the cell apoptosis rate in Xenograft tumor. *n* = 3. *P < 0.05, **P < 0.01, na: no statistical significance. Osimertinib Sensitive; OR: Osimertinib Resistance. RT-qPCR, reverse transcription-quantitative PCR. circ_100696: circ_PPAPDC1A. OE: OverExpression

The mouse xenograft tumor model presented in Fig. 9A was established to investigate the Osimertinib-sensitizing effects of circ_PPAPDC1A in vivo. Subsequently, 1 × 10^6^ Osimertinib resistance PC9/OR and HCC827/OR cells, which had been transfected with lentiviral vectors for control, circ_PPAPDC1A overexpression, or circ_PPAPDC1A knockdown, were subcutaneously injected into the armpits of nude mice. Comparative analysis with the control group revealed significant increases in tumor growth rate, tumor weight, and tumor volume in the circ_PPAPDC1A overexpressing group, whereas these parameters were notably reduced in the sh-circ_PPAPDC1A group (*P* < 0.05; Fig. 9B, C). Ki-67 staining was employed to assess the proliferative index of PC9/OR and HCC827/OR cells in vivo. The results indicated a significant increase in the percentage of Ki-67-positive cells in the circ_PPAPDC1A overexpressing group and a decrease in the sh-circ_PPAPDC1A group (*P* < 0.05; Fig. 9D). Additionally, TUNEL apoptosis assays conducted with xenograft tumor samples demonstrated that circ_PPAPDC1A overexpression inhibited apoptosis in PC9/OR cells in vivo, while circ_PPAPDC1A silencing promoted apoptosis in HCC827/OR cells in vivo (*P* < 0.05; Fig. 9F). These findings are consistent with the in vitro results, suggesting that circ_PPAPDC1A plays a role in suppressing Osimertinib sensitivity in NSCLC in vivo.

## Discussion

In the realm of cancer-associated circRNAs, abnormal circRNAs expression is linked to cancer cell invasion, migration, and metastasis, making them potential targets for diagnosis and therapy of cancer. Studies [26] have indicated that CircHERC1 promotes NSCLC progression by sequestering FOXO1 in the cytoplasm and regulating the miR-142-3p-HMGB1 axis. Moreover, circEPB41L2 blocks the progression and metastasis in non-small cell lung cancer by promoting TRIP12-triggered PTBP1 ubiquitylation [31]. Additionally, research has shown that CircSATB2 and miR-150-5p are regulators of TRIM66 in the regulation of growth and metastasis of NSCLC cells via the ceRNA pathway [32]. These findings collectively indicate that circRNAs can serve as oncogenes and tumor suppressors in NSCLC. Recent studies have also indicated that circRNAs are involved in the development and progression of resistance to first and second-generation EGFR-TKIs in NSCLC. WEN [33] identified significant differences in circRNAs expression in gefitinib- resistant cell lines through circRNAs sequencing. ZHU [34] found that circPSMC3 regulates PTEN expression by influencing the level of miR-10a-5p, thereby restoring sensitivity to gefitinib. ZHOU [35] further found that hcirc_0004015 can act as a sponge for miR-1183, influencing PDPK1 expression to promote gefitinib resistance. YANG [36], were observed significant upregulation of circ_102481 in exosomes of EGFR-TKIs-resistant patients. circ_102481 can promote EGFR-TKIs resistance through the miR-30a-5p/ROR1 axis in NSCLC. However, the extensive array of circRNAs and their specific functions in Osimertinib resistance of NSCLC continue to offer fertile ground for exploration and investigation.

In our study, we identified a circRNA closely associated with Osimertinib resistance: circ_PPAPDC1A. Initially, circ_PPAPDC1A was found to be lowly expressed or not expressed in human normal lung epithelial cells. However, with the onset of NSCLC, circ_PPAPDC1A showed a significant increase of 3-4-fold in NSCLC cells PC9 and HCC827, although this elevation was shown to be insufficient to affect the proliferation and growth of PC9 and HCC827 cells. Further investigation revealed that with the development of Osimertinib resistance, compared to PC9 and HCC827 cells, circ_PPAPDC1A significantly increased by 9-11-fold in Osimertinib resistance PC9/OR and HCC827/OR cells, surpassing the initial elevation observed during NSCLC initiation. Subsequent experiments also provided ample evidence that circ_PPAPDC1A can decrease the sensitivity of NSCLC cells to Osimertinib both in vitro and in vivo, acting as a “promoter of Osimertinib resistance”. circ_PPAPDC1A is located on chr 10 and belongs to the category of exonic circRNAs. Currently, there is limited research on circ_PPAPDC1A in the literature. William R [37] reported that circ_PPAPDC1A is abundantly expressed in human fibroblasts and is associated with ALU repetitive sequences. Agnieszka [38] also found that circ_PPAPDC1A is highly conserved, with dynamic expression in the mammalian brain. However, the functional studies of circ_PPAPDC1A and its regulatory roles in diseases such as cancer have not been explored. The coding gene for circ_PPAPDC1A is Phosphatidic Acid Phosphatase Type 2 Domain Containing 1 A (PPAPDC1A). PPAPDC1A is an essential diphosphatase gene located on human chr 10q26.12. It encodes an integral membrane phosphatase containing 271 amino acids and is a significant member of the PAP family. PPAPDC1A plays a crucial role in the activation of proteins like Protein Kinase C (PKC) and Tyrosine Protein Kinase (TPK), contributing to lipid metabolism and cell signal transduction pathways. Current research [39] has shown that PPAPDC1A is highly expressed in various tumors and acts as an oncogene, promoting tumor initiation and development. However, there is no existing research on the role of PPAPDC1A gene in EGFR-TKIs resistance in NSCLC. The encoding of circ_PPAPDC1A by the PPAPDC1A gene might be one of the crucial mechanisms through which it exerts its oncogenic effects. As far as we know, this is the first such report for the oncogene role of circ_PPAPDC1A in NSCLC with Osimertinib resistance.

Moreover, our findings indicate that circ_PPAPDC1A can interact with miR-30a-3p to influence the expression and activity of IGF1R. Recently, miR-30a-3p has emerged as the most prominent member of the miR-30a family. It originates from the 3’ arm of the miR-30a precursor and is located on chr 6q13. Current research indicates that miR-30a-3p acts as a tumor suppressor in the development of cancer [40]. Findings from our study were consistent with those in previous studies and further revealed that miR-30a-3p, acting as a tumor suppressor, was directly sponged and downregulated by circ_PPAPDC1A, with upregulation of IGF1R and activation of IGF1R/PI3K/ AKT/mTOR signaling. However, there are also study [41] indicates that LINC01436 promotes NSCLC progression by functioning as a miR-30a-3p sponge to regulate the expression of its target gene EPAS1.However, our study indicates that there is no significant difference in the expression of EPAS1 in Osimertinib resistance cells and it has no significant impact on the sensitivity to Osimertinib. So, we believe that EPAS1 may be involved in the occurrence of NSCLC through miR-30a-3p, but its impact on Osimertinib resistance is minimal. But more comprehensive research results may be needed to draw this conclusion definitively. Furthermore, we observed a progressive decrease in the expression of miR-30a-3p during the occurrence and development of NSCLC. Compared to adjacent normal tissues, miR-30a-3p decreased by 2-fold, but in Osimertinib resistance tissues, it decreased by over 10-fold. Therefore, we believe that miR-30a-3p is a crucial tumor suppressor in NSCLC, and cancer cells promote the occurrence, development, and resistance of NSCLC by inhibiting the expression of miR-30a-3p to affect the corresponding target genes.

Insulin-like growth factor-1 receptor (IGF1R) is a transmembrane tyrosine-protein kinase (TPK) receptor that primarily binds with the ligand insulin-like growth factor-1 (IGF-1). This binding trigger conformational changes and autophosphorylation of specific tyrosine residues, initiating signal transduction pathways that play a crucial role in cell proliferation, adhesion, transformation, and apoptosis [41]. Current research [42] indicates that IGF-1R is highly expressed in various tumors and functions as an oncogene in the development and progression of cancer. In particular, it has been reported previously that activation of IGF1R signaling is implicated in NSCLC differentiation and survival in patients, conferring acquired resistance to Osimertinib in NSCLC containing the EGFR T790M mutation [43]. Many strategies to target IGF1R signaling have been developed, including siRNA and monoclonal antibodies for IGF1R and kinase inhibitors to inhibit the enzyme activity of RTK. PI3K/AKT/ mTOR pathway are typical signal transduction pathways that are crucial for first/second-generation EGFR-TKIs and third-generation EGFR-TKIs such as Osimertinib resistance events in NSCLC, and also defined as downstream of the IGF1R signaling pathways. Although Osimertinib resistance is more complex, much evidence has implied that activation of the PI3K/AKT/mTOR pathway is required for Osimertinib resistance in NSCLC (EGFR Bypass Activation Pathway) [44]. Our study confirms that miR-30a-3p is an important upstream regulator of IGF1R. MiR-30a-3p can specifically bind to IGF1R and significantly inhibit the expression of IGF1R. MiRNA regulation is likely a crucial factor in the activation of IGF1R. We also found that overexpression of circ_PPAPDC1A activates the PI3K/AKT/mTOR signaling pathway, while inhibiting circ_PPAPDC1A can suppress the PI3K/AKT/mTOR signaling pathway. MiR-30a-3p can restore the regulation of circ_PPAPDC1A on the PI3K/AKT/mTOR signaling pathway. Furthermore, this provides further evidence that IGF1R can be involved in NSCLC Osimertinib resistance through the PI3K/ AKT/mTOR signaling pathway.

## Conclusion

In conclusion, for the first time we emphasized and identified that circ_PPAPDC1A was significantly upregulated and exerts an oncogenic role in Osimertinib resistance of NSCLC by sponging miR-30a-3p to active IGF1R/PI3K/AKT/mTOR pathway in NSCLC. circ_PPAPDC1A may serve as a novel diagnostic biomarker and therapeutic target for NSCLC patients with Osimertinib resistance. Our findings highlight that the investigation of circRNAs and their role in Osimertinib resistance in NSCLC represents a promising research direction. As our comprehension of the complex regulatory functions of circRNAs advances, it holds the potential to unveil novel strategies for enhancing the efficacy of targeted therapies and surmounting drug resistance in NSCLC patients. Further research and clinical validation are essential to fully harness the clinical significance of circRNAs in this context.

### Supplementary Information

**Additional file 1:** **Supplemental Figure 1.** Differential expression of circRNAs in OR and OS tissues. (A) circRNA microarray hybridization signal diagram; (B) Distribution of differentially expressed circRNAs; (C) circRNAs Volcano plots. The red dot represents the up-regulated circRNAs, and the green dot represents the down-regulated circRNAs; (D) circRNAs Scatter plot. The X-axis and Y-axis are the standardized signals. OS: Osimertinib Sensitive; OR: Osimertinib Resistance. **Supplemental Figure 2. **(A) Chromosome location, exon splicing site and pattern map of circRNA_100696. circ_100696 was located on chromosome 10 at position q26.12: 122273422-122280607 and formed by the splicing of the 3rd, 4th, and 5th exons of the PPAPDC1A gene. (B) Fluorescence in situ hybridization assay revealed that circ_100696 is widely present in PC9/OR and HCC827/OR cells, with the primary localization being enriched in the cytoplasm. OS: Osimertinib Sensitive; OR: Osimertinib Resistance. circ_100696: circ_PPAPDC1A. **Supplemental Figure 3.** (A) circ_PPAPDC1A exhibits significantly lower expression in human normal lung epithelial cells EAS-2B compared to PC9 and HCC827 cells. (B) CCK-8 assay showed that circ_PPAPDC1A has no significant effect on the cell proliferation of PC9 and HCC827 cells. n=3. *P<0.05, na: no statistical significance. Osimertinib Sensitive; OR: Osimertinib Resistance. RT-qPCR, reverse transcription- quantitative PCR. circ_100696: circ_PPAPDC1A. OE: OverExpression. **Supplemental Figure 4. **Pearson's correlation analysis**. **(A) Pearson's correlation analysis highlighted a negative correlation trend between circ_PPAPDC1A and miR-30a-3p expression (*n*=5; *r*=-0.81, *P*=0.11). (B) Pearson's correlation analysis highlighted a negative correlation trend between IGF1R mRNA and miR-30a-3p expression in Osimertinib resistance tissues (*n*=5; *r*=-0.74,*P*=0.15; Supplemental Figure 3B). Although there was no statistical significance due to the small sample size. **Supplemental Figure 5. **The role of EPAS1 in Osimertinib resistance of NSCLC. (A) EPAS1 mRNA expression in NSCLC Osimertinib sensitive and resistance cells was assessed by RT-qPCR. (B) EPAS1 protein expression in NSCLC Osimertinib sensitive and resistance cells was assessed by Western Blot. (C, D) Cell counting kit-8 (CCK8) assay was conducted to determine the sensitivity of PC9, HCC829, PC9/OR, and HCC829/OR cells to Osimertinib. *n*=3. #: no statistical significance. Osimertinib Sensitive; OR: Osimertinib Resistance. RT-qPCR, reverse transcription-quantitative PCR.

**Additional file 2:** **Supplemental Table 1.** Clinical pathological materials of 5 cases with Osimertinib resistance. **Supplemental Table 2. **Concentration and purity of total RNA in each sample (*N*=10). **Supplemental Table 3. **Efficiency of RNA labeling in each samples (*N*=10). **Supplemental Table 4. **Top 10 GO BP categories for DE circRNA coding genes. **Supplemental Table 5. **Top 10 GO CC categories for DE circRNA coding genes. **Supplemental Table 6. **Top 10 GO MF categories for DE circRNA coding genes. **Supplemental Table 7. **Top 10 KEGG signaling pathways for DE circRNA coding genes. **Supplemental Table 9.** The Western blot antibody information. **Supplemental Table 10.** Relative expression of IGF1R/PI3K/AKT/mTOR pathway. **Supplemental Table 11.** Relative expression of IGF1R/PI3K/AKT/mTOR pathway.

## Data Availability

The datasets used and/or analyzed during the current study are available from the corresponding author on reasonable request.
