# Supplementary material for: circ_PPAPDC1A promotes Osimertinib resistance by sponging the miR-30a-3p/ IGF1R pathway in non-small cell lung cancer (NSCLC)
Source: Mol Cancer. 2024 May 7;23:91. doi: 10.1186/s12943-024-01998-w (PMC11075361; doi:10.1186/s12943-024-01998-w)
Supplement: Supplementary file 1 — Additional file 1: Supplemental Figure 1. Differential expression of circRNAs in OR and OS tissues. (A) circRNA microarray hybridization signal diagram; (B) Distribution of differentially expressed circRNAs; (C) circRNAs Volcano plots. The red dot represents the up-regulated circRNAs, and the green dot represents the down-regulated circRNAs; (D) circRNAs Scatter plot. The X-axis and Y-axis are the standardized signals. OS: Osimertinib Sensitive; OR: Osimertinib Resistance. Supplemental Figure 2. (A) Chromosome location, exon splicing site and pattern map of circRNA_100696. circ_100696 was located on chromosome 10 at position q26.12: 122273422-122280607 and formed by the splicing of the 3rd, 4th, and 5th exons of the PPAPDC1A gene. (B) Fluorescence in situ hybridization assay revealed that circ_100696 is widely present in PC9/OR and HCC827/OR cells, with the primary localization being enriched in the cytoplasm. OS: Osimertinib Sensitive; OR: Osimertinib Resistance. circ_100696: circ_PPAPDC1A. Supplemental Figure 3. (A) circ_PPAPDC1A exhibits significantly lower expression in human normal lung epithelial cells EAS-2B compared to PC9 and HCC827 cells. (B) CCK-8 assay showed that circ_PPAPDC1A has no significant effect on the cell proliferation of PC9 and HCC827 cells. n=3. *P<0.05, na: no statistical significance. Osimertinib Sensitive; OR: Osimertinib Resistance. RT-qPCR, reverse transcription- quantitative PCR. circ_100696: circ_PPAPDC1A. OE: OverExpression. Supplemental Figure 4. Pearson's correlation analysis. (A) Pearson's correlation analysis highlighted a negative correlation trend between circ_PPAPDC1A and miR-30a-3p expression (n=5; r=-0.81, P=0.11). (B) Pearson's correlation analysis highlighted a negative correlation trend between IGF1R mRNA and miR-30a-3p expression in Osimertinib resistance tissues (n=5; r=-0.74,P=0.15; Supplemental Figure 3B). Although there was no statistical significance due to the small sample size. Supplemental Figure 5. The role of E [file 12943_2024_1998_MOESM1_ESM.docx]

**Supplemental Figures and legends**


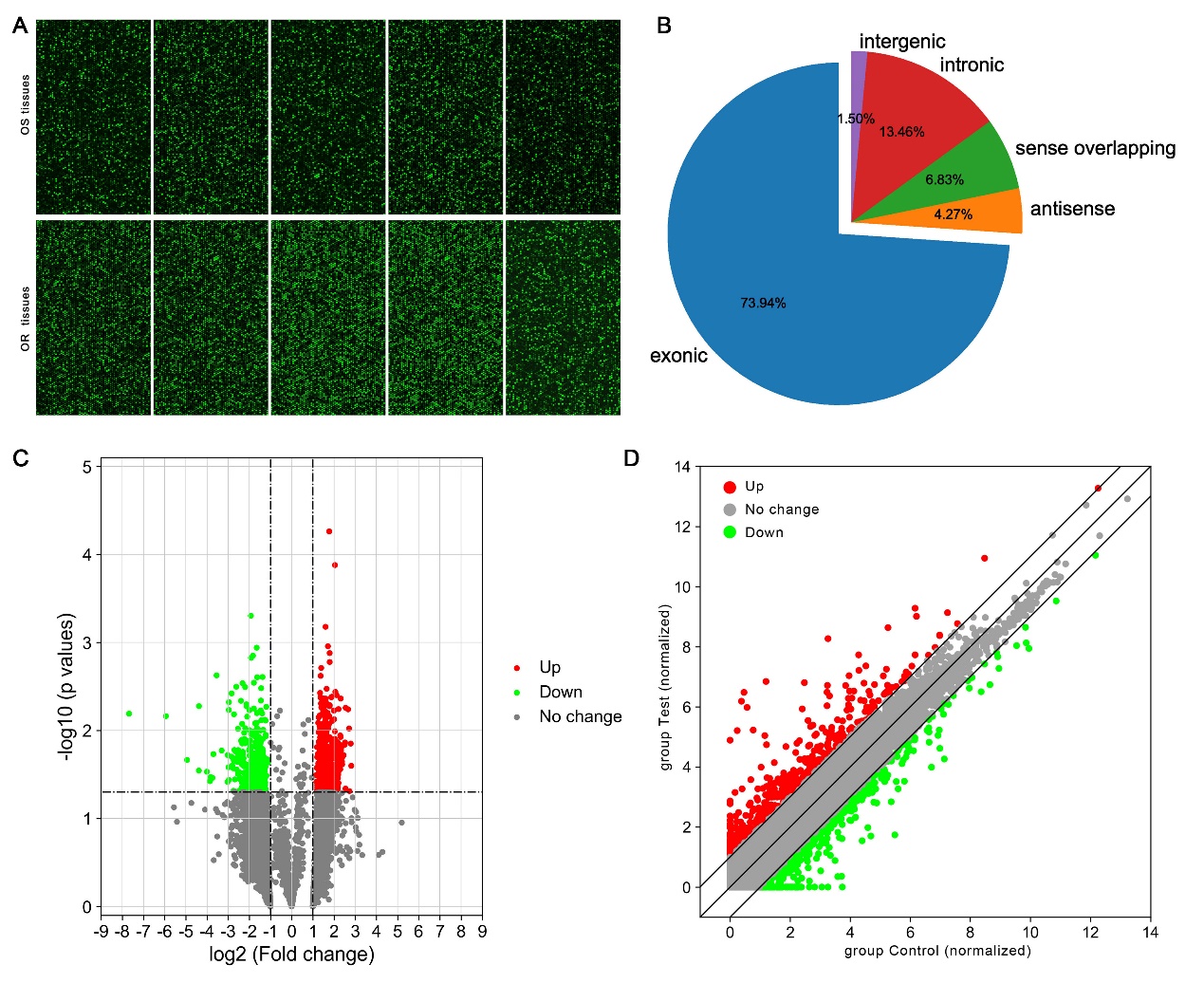


**Supplemental Figure 1.** Differential expression of circRNAs in OR and OS tissues. (A) circRNA microarray hybridization signal diagram; (B) Distribution of differentially expressed circRNAs; (C) circRNAs Volcano plots. The red dot represents the up-regulated circRNAs, and the green dot represents the down-regulated circRNAs; (D) circRNAs Scatter plot. The X-axis and Y-axis are the standardized signals. OS: Osimertinib Sensitive; OR: Osimertinib Resistance.


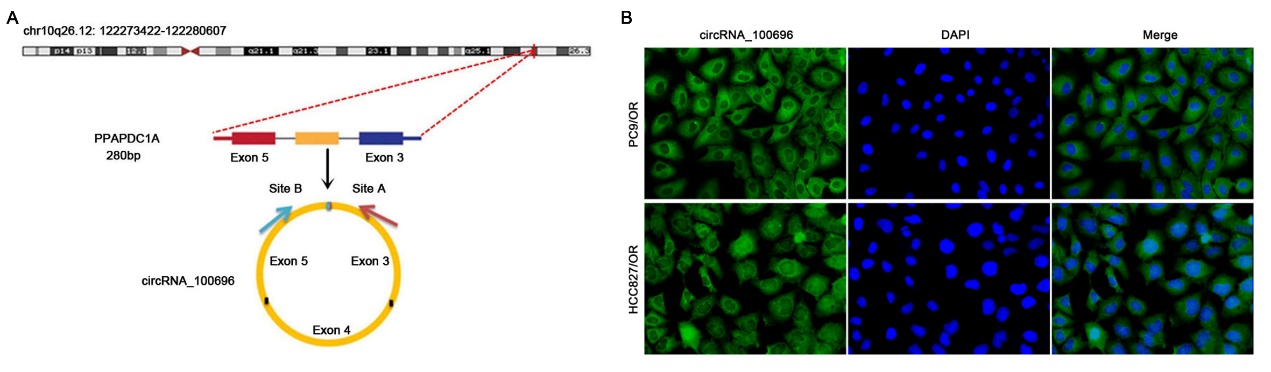


**Supplemental Figure 2.** (A) Chromosome location, exon splicing site and pattern map of circRNA_100696. circ_100696 was located on chromosome 10 at position q26.12: 122273422-122280607 and formed by the splicing of the 3rd, 4th, and 5th exons of the PPAPDC1A gene. (B) Fluorescence in situ hybridization assay revealed that circ_100696 is widely present in PC9/OR and HCC827/OR cells, with the primary localization being enriched in the cytoplasm. OS: Osimertinib Sensitive; OR: Osimertinib Resistance. circ_100696: circ_PPAPDC1A.


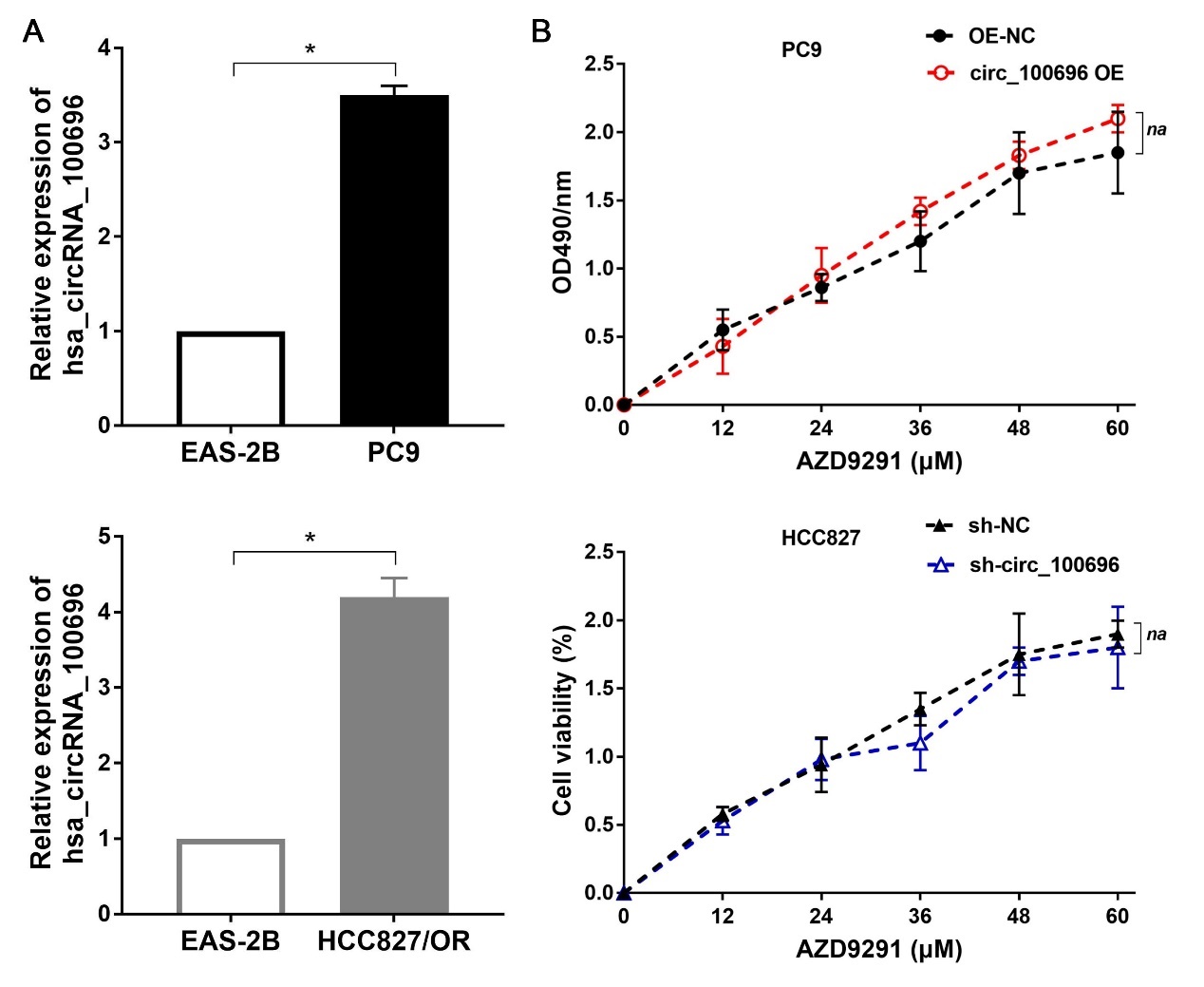


**Supplemental Figure 3.** (A) circ_PPAPDC1A exhibits significantly lower expression in human normal lung epithelial cells EAS-2B compared to PC9 and HCC827 cells. (B) CCK-8 assay showed that circ_PPAPDC1A has no significant effect on the cell proliferation of PC9 and HCC827 cells. n=3. *P<0.05, na: no statistical significance. Osimertinib Sensitive; OR: Osimertinib Resistance. RT-qPCR, reverse transcription- quantitative PCR. circ_100696: circ_PPAPDC1A. OE: OverExpression.


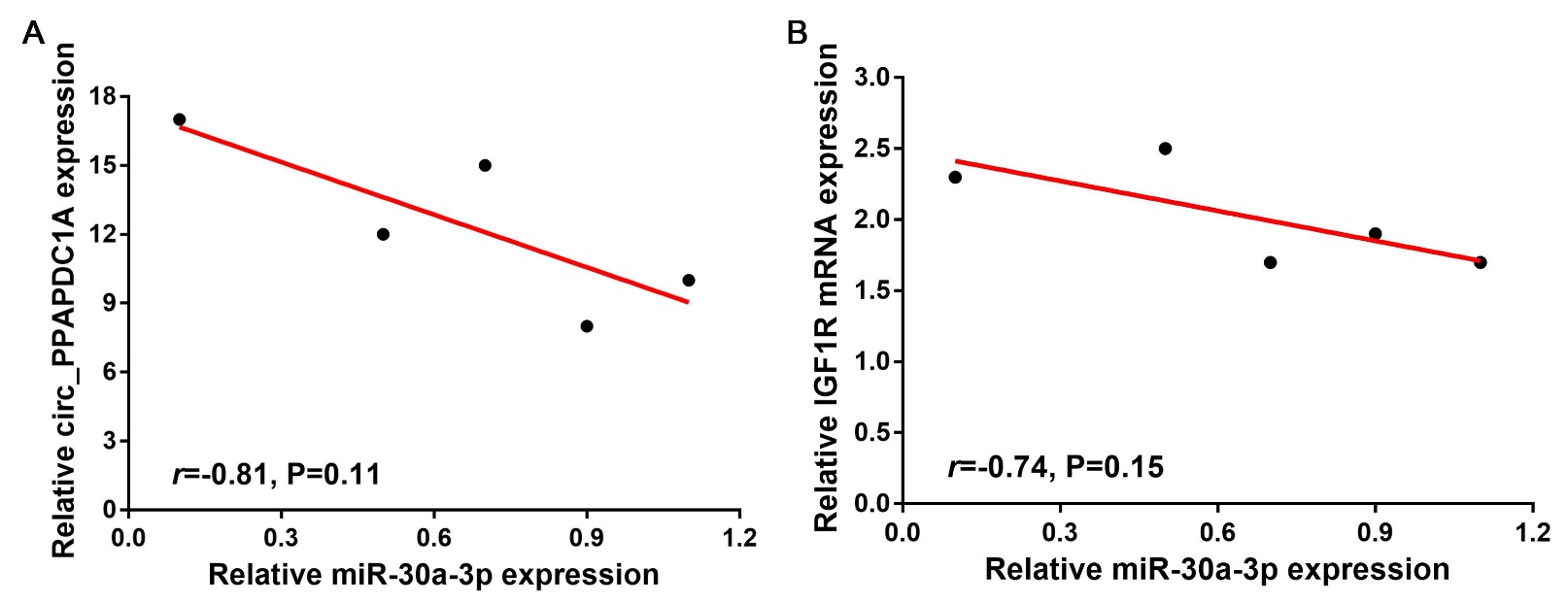


**Supplemental Figure 4.** Pearson's correlation analysis**.** (A) Pearson's correlation analysis highlighted a negative correlation trend between circ_PPAPDC1A and miR-30a-3p expression (n=5; r=-0.81, P=0.11). (B) Pearson's correlation analysis highlighted a negative correlation trend between IGF1R mRNA and miR-30a-3p expression in Osimertinib resistance tissues (n=5; r=-0.74, P=0.15; Supplemental Figure 3B). Although there was no statistical significance due to the small sample size.


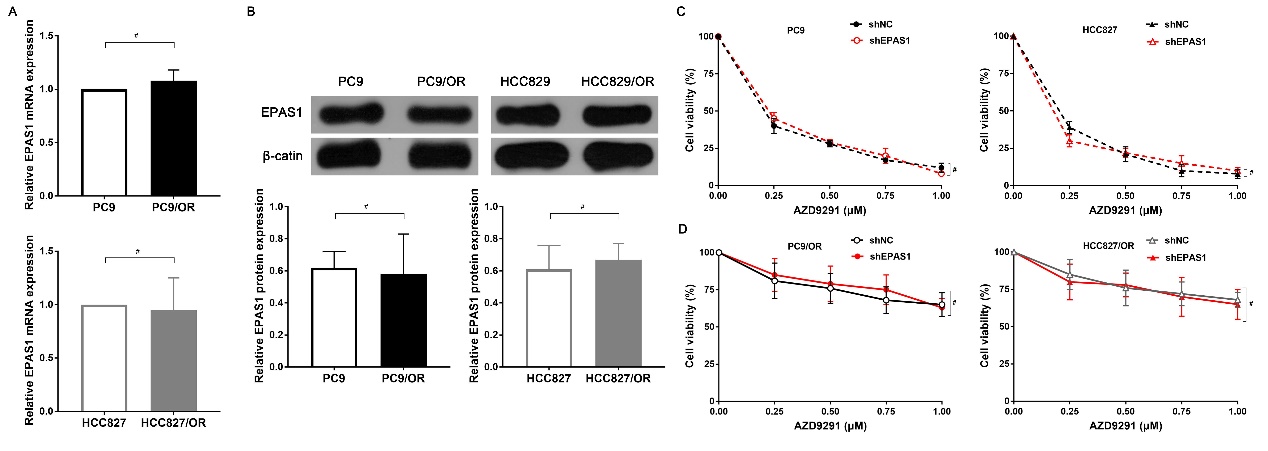


**Supplemental Figure 5.** The role of EPAS1 in Osimertinib resistance of NSCLC. (A) EPAS1 mRNA expression in NSCLC Osimertinib sensitive and resistance cells was assessed by RT-qPCR. (B) EPAS1 protein expression in NSCLC Osimertinib sensitive and resistance cells was assessed by Western Blot. (C-D) Cell counting kit-8 (CCK8) assay was conducted to determine the sensitivity of PC9, HCC829, PC9/OR, and HCC829/OR cells to Osimertinib. n=3. #: no statistical significance. Osimertinib Sensitive; OR: Osimertinib Resistance. RT-qPCR, reverse transcription-quantitative PCR.
