# Supplementary material for: circ_PPAPDC1A promotes Osimertinib resistance by sponging the miR-30a-3p/ IGF1R pathway in non-small cell lung cancer (NSCLC)
Source: Mol Cancer. 2024 May 7;23:91. doi: 10.1186/s12943-024-01998-w (PMC11075361; doi:10.1186/s12943-024-01998-w)
Supplement: Supplementary file 2 — Additional file 2: Supplemental Table 1. Clinical pathological materials of 5 cases with Osimertinib resistance. Supplemental Table 2. Concentration and purity of total RNA in each sample (N=10). Supplemental Table 3. Efficiency of RNA labeling in each samples (N=10). Supplemental Table 4. Top 10 GO BP categories for DE circRNA coding genes. Supplemental Table 5. Top 10 GO CC categories for DE circRNA coding genes. Supplemental Table 6. Top 10 GO MF categories for DE circRNA coding genes. Supplemental Table 7. Top 10 KEGG signaling pathways for DE circRNA coding genes. Supplemental Table 9. The Western blot antibody information. Supplemental Table 10. Relative expression of IGF1R/PI3K/AKT/mTOR pathway. Supplemental Table 11. Relative expression of IGF1R/PI3K/AKT/mTOR pathway. [file 12943_2024_1998_MOESM2_ESM.docx]

**Supplemental Tables**

**Supplemental Table 1.** Clinical pathological materials of 5 cases with Osimertinib resistance

|  | **Age** | **Gender** | **Histopathology** | **Pathological Grading** | **TNM Staging** | **Pre-resistance**  **Molecular Typing** | **Post-resistance Molecular Typing** |
| --- | --- | --- | --- | --- | --- | --- | --- |
| Case_1 | 56 | Female | Adenocarcinoma | Low | IVB | T790M mutation | C797S point mutation |
| Case_2 | 51 | Male | Adenocarcinoma | Middle | IVB | T790M mutation | C797S point mutation |
| Case_3 | 48 | Female | Adenocarcinoma | Low | IVB | T790M mutation | c-MET amplification |
| Case_4 | 49 | Female | Adenocarcinoma | Low | IVB | T790M mutation | c-MET amplification |
| Case_5 | 62 | Female | Adenocarcinoma | High | IVB | T790M mutation | T790M deletion |

**Supplemental Table 2.** Concentration and purity of total RNA in each sample (N=10)

| Samples | A260/280 value | A260/230 value | Concentration(ng /μL) | Volume（μL） | Quality（ng） |
| --- | --- | --- | --- | --- | --- |
| OS^a^_1 | 2.01 | 2.08 | 780 | 60 | 46800 |
| OS_2 | 2.03 | 2.04 | 690 | 80 | 55200 |
| OS_3 | 1.90 | 2.12 | 960 | 50 | 48000 |
| OS_4 | 2.04 | 2.01 | 620 | 100 | 62000 |
| OS_5 | 1.95 | 2.12 | 650 | 120 | 78000 |
| OR^b^_1 | 2.08 | 2.06 | 780 | 80 | 62400 |
| OR_2 | 1.96 | 2.08 | 810 | 80 | 64800 |
| OR_3 | 1.98 | 2.11 | 860 | 60 | 51600 |
| OR_4 | 2.05 | 2.15 | 1100 | 50 | 55000 |
| OR_5 | 1.98 | 2.13 | 650 | 90 | 58500 |

^a^OS: Osimertinib Sensitive, ^b^OR: Osimertinib Resistance.

**Supplemental Table 3.** Efficiency of RNA labeling in each samples(N=10)

|  | Dye Name | Dye (pmol/μL) | cRNA Concentration (μg/μL） | Specific Activity^c^(pmol Dye/μg cRNA) | Vol(μL） | Total Amount (μg） |
| --- | --- | --- | --- | --- | --- | --- |
| OS_1 | cy3 | 13.54 | 0.75 | 18.05 | 20.00 | 15.00 |
| OS_2 | cy3 | 12.56 | 0.77 | 16.31 | 20.00 | 15.40 |
| OS_3 | cy3 | 13.58 | 0.81 | 16.77 | 20.00 | 16.20 |
| OS_4 | cy3 | 10.29 | 0.86 | 11.97 | 20.00 | 17.20 |
| OS_5 | cy3 | 11.23 | 0.78 | 14.40 | 20.00 | 15.60 |
| OR_1 | cy3 | 10.89 | 0.82 | 13.28 | 20.00 | 16.40 |
| OR_2 | cy3 | 14.12 | 0.76 | 18.58 | 20.00 | 15.20 |
| OR_3 | cy3 | 11.35 | 0.78 | 14.55 | 20.00 | 15.60 |
| OR_4 | cy3 | 10.56 | 0.82 | 12.89 | 20.00 | 16.40 |
| OR_5 | cy3 | 12.45 | 0.83 | 15.00 | 20.00 | 16.60 |

^c^Specific Activity = (pmol per μL dye)/ (μg per μL cRNA)

**Supplemental Table 4.** Top 10 GO BP categories for DE circRNA coding genes.

| GO.ID | GO-BP Term | p value |
| --- | --- | --- |
| GO:0050794 | regulation of cellular process | 2.03E-06 |
| GO:0007154 | cell communication | 1.08E-05 |
| GO:0031056 | regulation of histone modification | 4.16E-05 |
| GO:0065007 | biological regulation | 2.35E-04 |
| GO:0051276 | chromosome organization | 5.85E-04 |
| GO:0043487 | regulation of RNA stability | 2.32E-03 |
| GO:0014033 | neural crest cell differentiation | 5.87E-03 |
| GO:0043484 | regulation of RNA splicing | 8.45E-03 |
| GO:0009887 | organ morphogenesis | 1.17E-02 |
| GO:0016070 | RNA metabolic process | 3.45E-02 |

GO: Gene Ontology, BP: Biological Process, DE: differentially expressed.

**Supplemental Table 5.** Top 10 GO CC categories for DE circRNA coding genes.

| GO.ID | GO-CC Term | p value |
| --- | --- | --- |
| GO:0030054 | cell junction | 3.12E-06 |
| GO:0043227 | membrane-bounded organelle | 6.05E-06 |
| GO:0044424 | intracellular part | 1.21E-05 |
| GO:0005942 | phosphatidylinositol 3-kinase complex | 3.45E-05 |
| GO:0070603 | SWI/SNF superfamily-type complex | 8.43E-05 |
| GO:0000228 | nuclear chromosome | 2.35E-04 |
| GO:0005875 | microtubule associated complex | 6.46E-04 |
| GO:0005815 | microtubule-organizing center | 2.15E-03 |
| GO:0070013 | intracellular organelle lumen | 4.34E-03 |
| GO:1904949 | ATPase complex | 2.16E-02 |

GO: Gene Ontology, CC: Cellular component, DE: differentially expressed.

**Supplemental Table 6.** Top 10 GO MF categories for DE circRNA coding genes.

| GO.ID | GO-MF Term | p value |
| --- | --- | --- |
| GO:0003723 | RNA binding | 1.15E-05 |
| GO:0042803 | protein homodimerization activity | 5.76E-05 |
| GO:0008094 | DNA-dependent ATPase activity | 6.43E-05 |
| GO:0019904 | protein domain specific binding | 8.26E-05 |
| GO:0008301 | DNA binding, bending | 1.46E-04 |
| GO:0003730 | mRNA 3'-UTR binding | 6.37E-04 |
| GO:0008134 | transcription factor binding | 2.26E-03 |
| GO:0003682 | chromatin binding | 5.49E-03 |
| GO:0019208 | phosphatase regulator activity | 2.41E-02 |
| GO:0001105 | RNA polymerase II transcription activity | 4.69E-02 |

GO: Gene Ontology, MF: Molecular Function, DE: differentially expressed.

**Supplemental Table 7.** Top 10 KEGG signaling pathways for DE circRNA coding genes

| KEGG ID | KEGG Name | p value |
| --- | --- | --- |
| hsa04350 | TGF-beta signaling pathway | 3.14E-03 |
| hsa05291 | IGF-II signaling pathway | 5.34E-03 |
| hsa04310 | Wnt signaling pathway | 6.15E-03 |
| hsa04390 | Hippo signaling pathway | 8.12E-03 |
| hsa05200 | Pathways in cancer | 8.15E-03 |
| hsa04390 | Hippo signaling pathway | 1.35E-02 |
| hsa05200 | Pathways in cancer | 3.24E-02 |
| hsa04340 | Hedgehog signaling pathway | 5.24E-02 |
| hsa05205 | Proteoglycans in cancer | 5.72E-02 |
| hsa04340 | Hedgehog signaling pathway | 8.16E-02 |

KEGG: Kyoto Encyclopedia of Genes and Genomes, DE: differentially expressed.

**Supplemental Table 8.** The spliced sequence of circ_100696

| circRNA | Splicing sequence |
| --- | --- |
| circ_100696 | 5’GCAATTTCTTTCCTCACACCCCTGGCTGTTATTTGTGTGGTGAAAATTATCCGGCGAACAGACAAGACTGAAATTAAGGAAGCCTTCTTAGCGGTGTCCTTGGCTCTTGCTTTGAATGGAGTCTGCACAAACACTATTAAATTAATAGTGGGAAGACCTCGCCCCGATTTCTTTTACCGCTGCTTTCCAGATGGAGTGATGAACTCGGAAATGCATTGCACAGGTGACCCCGATCTGGTGTCCGAGGGCCGCAAAAGCTTCCCCAGCATCCATTCCTCCT-3’ |

**Supplemental Table 9.** The Western blot antibody information.

| Antibody | Animal Source | Dilution Ratio | Storage Condition | Company | Batch Number |
| --- | --- | --- | --- | --- | --- |
| IGF1R | Rabbit IgG | 1:1000 | 4℃，Avoid light | Abcam | ab182408 |
| PI3K | Rabbit IgG | 1:1000 | 4℃，Avoid light | Abcam | ab32089 |
| p-PI3K | Rabbit IgG | 1:500 | 4℃，Avoid light | Abcam | ab278545 |
| AKT | Rabbit IgG | 1:1000 | 4℃，Avoid light | Abcam | ab64148 |
| p-AKT | Rabbit IgG | 1:500 | 4℃，Avoid light | Abcam | ab38449 |
| mTOR | Rabbit IgG | 1:1000 | 4℃，Avoid light | Abcam | ab134903 |
| p-mTOR | Rabbit IgG | 1:1000 | 4℃，Avoid light | Abcam | ab109268 |
| HRP secondary antibody | Goat IgG | 1:5000 | 4℃，Avoid light | Abcam | ab6721 |

**Supplemental Table 10.** Relative expression of IGF1R/PI3K/AKT/mTOR pathway.

|  | OE-NC | circ_100696 OE | miR-30a-3p mimic | OE+mimic |
| --- | --- | --- | --- | --- |
| PI3K | 0.11 | 0.41 | 0.04 | 0.22 |
| p-PI3K | 0.59 | 0.56 | 0.53 | 0.54 |
| AKT | 0.15 | 0.46 | 0.05 | 0.41 |
| p-AKT | 0.67 | 0.58 | 0.63 | 0.56 |
| mTOR | 0.19 | 0.42 | 0.11 | 0.34 |
| p-mTOR | 0.61 | 0.61 | 0.60 | 0.57 |
| IGF1R | 0.21 | 0.53 | 0.12 | 0.38 |

**Supplemental Table 11.** Relative expression of IGF1R/PI3K/AKT/mTOR pathway.

|  | sh-NC | sh-circ_100696 | miR-30a-3p inhibitor | sh+inhibitor |
| --- | --- | --- | --- | --- |
| IGF1R | 0.26 | 0.13 | 0.58 | 0.41 |
| PI3K | 0.71 | 0.63 | 0.71 | 0.70 |
| p-PI3K | 0.34 | 0.13 | 0.55 | 0.30 |
| AKT | 0.67 | 0.60 | 0.78 | 0.81 |
| p-AKT | 0.36 | 0.08 | 0.59 | 0.26 |
| mTOR | 0.63 | 0.61 | 0.69 | 0.78 |
| p-mTOR | 0.51 | 0.17 | 0.57 | 0.47 |
